# A Royal Road to Quantum Theory (or Thereabouts)

**DOI:** 10.3390/e20040227

**Published:** 2018-03-26

**Authors:** Alexander Wilce

**Affiliations:** Department of Mathematics, Susquehanna University, Selinsgrove, PA 17870, USA; wilce@susqu.edu

**Keywords:** reconstruction of quantum mechanics, conjugate systems, Jordan algebras

## Abstract

This paper fails to derive quantum mechanics from a few simple postulates. However, it gets very close, and does so without much exertion. More precisely, I obtain a representation of finite-dimensional probabilistic systems in terms of Euclidean Jordan algebras, in a strikingly easy way, from simple assumptions. This provides a framework within which real, complex and quaternionic QM can play happily together and allows some (but not too much) room for more exotic alternatives. (This is a leisurely summary, based on recent lectures, of material from the papers arXiv:1206:2897 and arXiv:1507.06278, the latter joint work with Howard Barnum and Matthew Graydon. Some further ideas are also explored, developing the connection between conjugate systems and the possibility of forming stable measurement records and making connections between this approach and the categorical approach to quantum theory.)

## 1. Introduction and Overview

Whatever else it may be, Quantum mechanics (QM) is a machine for making probabilistic predictions about the results of measurements. To this extent, QM is, at least in part, about information. Over the last decade or so, it has become clear that the formal apparatus of quantum theory, at least in finite dimensions, can be recovered from constraints on how physical systems store and process information. To this extent, finite-dimensional QM is just about information.

The broad idea of regarding QM in this way, and of attempting to derive its mathematical structure from simple operational or probabilistic axioms, is not new. Efforts in this direction go back at least to the work of von Neumann [1], and include also attempts by Schwinger [2], Mackey [3], Ludwig [4], Piron [5], and many others. However, the consensus is that these were not entirely successful: partly because the results they achieved (e.g., Piron’s well-known representation theorem) did not rule out certain rather exotic alternatives to QM, but mostly because the axioms deployed seem, in retrospect, to lack sufficient physical or operational motivation.

More recently, with inspiration from quantum information theory, attention has focused on finite-dimensional systems, where the going is a bit easier. Just as importantly, quantum information theory prompts us to treat properties of composite systems as fundamental, where earlier work focused largely on systems in isolation (a recent exception to this trend is the paper [6] of Barnum, Müller and Ududec). These shifts of emphasis are illustrated by the work of Hardy [7], who presented five simple, broadly information-theoretic postulates governing the states and measurements associated with a physical system, determining a very restricted set of possible theories, parametrized by a positive integer *r*, with finite-dimensional quantum and classical probability theory corresponding to r=1 and r=2. Following this lead, several papers, notably [8,9,10], have derived finite-dimensional QM from various packages of axioms governing the information-carrying and information-processing capacity of finite-dimensional systems.

**Problems with existing approaches.** These recent reconstructive efforts suffer from two related problems. First, they make use of assumptions that seem too strong. Secondly, in trying to derive exactly complex, finite-dimensional quantum theory, they derive too much.

- All of the cited papers assume local tomography. This is the doctrine that the state of a bipartite composite system is entirely determined by the joint probabilities it assigns to outcomes of measurements on the two subsystems. This rules out both real and quaternionic QM, both of which are legitimate quantum theories [11].
- These papers also all make some version of a uniformity assumption: that all systems having the same information-carrying capacity are isomorphic, or that all systems are composed, in a uniform way, from “bits” of a uniform type. Here, “information carrying capacity” means essentially the maximum number of states that can be distinguished from one another with probability one by a single measurement. A bit is a system for which this number is two. This rules out systems involving superselection rules, i.e., those that admit both real and classical degrees of freedom (for example, the quantum system corresponding to $M_{2}\left(C\right)⊕M_{2}\left(C\right)$, corresponding to a classical choice between one of two qubits, has the same information-carrying capacity as a single, four-level quantum system). More seriously, it rules out any theory that includes, e.g., real and complex, or real and quaternionic systems, as the state spaces of the bits of these theories have different dimensions. As I will discuss below, one can indeed construct mathematically-reasonable theories that embrace finite-dimensional quantum systems of all three types.
- Another shortcoming, not related to the exclusion of real and quaternionic QM, is the technical assumption (explicit in [10] for bits) that all positive affine functionals on the state space taking values between zero and one correspond to physically-accessible “effects”, i.e., possible measurement results. From an operational point of view, this principle (called the “no-restriction hypothesis” in [12]) seems to call for further motivation.

**Another approach.** In these notes, I am going to describe an alternative approach that avoids these difficulties. This begins by associating with every physical system a convex set of states and a distinguished set of basic measurements (or experiments) that can be made on the system. We then isolate two striking features shared by classical and quantum probabilistic systems. The first is the possibility of finding a joint state that perfectly correlates a system *A* with an isomorphic system $\bar{A}$ (call it a conjugate system) in the sense that every basic measurement on *A* is perfectly correlated with the corresponding measurement on $\bar{A}$. In finite-dimensional QM, where *A* is represented by a finite-dimensional Hilbert space H, $\bar{A}$, corresponds to the conjugate Hilbert space $\bar{H}$, and the perfectly-correlating state is the maximally-entangled “EPR” state on $H⊗\bar{H}$.

The second feature is the existence of what I call filters associated with each basic measurement. These are processes that independently attenuate the “response” of each outcome of the measurement by some specified factor. Such a process will generally not preserve the normalization of states, but up to a constant factor, in both classical and quantum theory, one can prepare any desired state by applying a suitable filter to the maximally-mixed state. Moreover, when the target state is not singular (that is, when it does not assign probability zero to any nonzero measurement outcome), one can reverse the filtering process, in the sense that it can be undone by another process with positive probability.

The upshot is that all probabilistic systems having conjugates and a sufficiently lavish supply of (probabilistically) reversible filters can be represented by formally real Jordan algebras, a class of structures that includes real, complex and quaternionic quantum systems, and just two further well-studied additional possibilities, which I will review below.

In addition to leaving room for real and quaternionic quantum mechanics (which I take to be a virtue), this approach has another advantage: it is much easier! The assumptions involved are few and easily stated, and the proof of the main technical result (Lemma 1 in Section 4) is short and straightforward. By contrast, the mathematical developments in the papers listed above are significantly more difficult and ultimately lean on the (even more difficult) classification of compact groups acting on spheres. My approach, too, leans on a received result, but one that is relatively accessible. This is the Koecher–Vinberg theorem, which characterizes formally real, or Euclidean, Jordan algebras in terms of ordered real vector spaces with homogeneous, self-dual cones. A short and non-taxing proof of this classical result can be found in [13].

These ideas were developed in [14,15,16] and especially [17], of which this paper is, to an extent, a summary. However, the presentation here is slightly different, and some additional ideas are also explored. In particular, I have spelled out in more detail the connection between conjugate systems and measurement records, only alluded to in the earlier paper. I also link this approach to the categorical approach to quantum theory due to Abramsky, Coecke and others [18], along the way briefly discussing recent work with Howard Barnum and Matthew Graydon [19] on the construction of probabilistic theories in which real, complex and quaternionic quantum systems coexist. Finally, Appendix B presents a uniqueness result for spectral decompositions of states, which may find further application.

**A bit of background.** At this point, I had better pause to explain some terms. A *Jordan algebra* is a real commutative algebra (a real vector space E with a commutative bilinear multiplication a,b↦a·b) having a multiplicative unit *u* and satisfying the Jordan identity: $a^{2}\cdot \left(a\cdot b\right)=a\cdot \left(a^{2}\cdot b\right)$, for all a,b,c∈E, where $a^{2}=a\cdot a$. A Jordan algebra is *formally real* if sums of squares of nonzero elements are always nonzero. The basic, and motivating, example is the space $L_{\mathrm{sa}}\left(H\right)$ of self-adjoint operators on a complex Hilbert space, with the Jordan product given by $a\cdot b=\frac{1}{2}\left(ab+ba\right)$. Note that here, a·a=aa, so the notation $a^{2}$ is unambiguous. To see that $L_{\mathrm{sa}}\left(H\right)$ is formally real, just note that $a^{2}$ is always a positive operator.

If H is finite dimensional, $L_{\mathrm{sa}}\left(H\right)$ carries a natural inner product, namely 〈a,b〉=Tr(ab). This plays well with the Jordan product: 〈a·b,c〉=〈b,a·c〉 for all $a,b,c\in L_{\mathrm{sa}}\left(H\right)$. More generally, a finite-dimensional Jordan algebra equipped with an inner product having this property is said to be *Euclidean*. For finite-dimensional Jordan algebras, being formally real and being Euclidean are equivalent [13]. In what follows, I will abbreviate “Euclidean Jordan algebra” to EJA.

Jordan algebras were originally proposed, with what now looks like slightly thin motivation, by P. Jordan [20]: if *a* and *b* are quantum-mechanical observables, represented by $a,b\in L_{\mathrm{sa}}\left(H\right)$, then while a+b is again self-adjoint, ab and ba are not, unless *a* and *b* commute; however, their average, a·b, is self-adjoint and, thus, represents another observable. Almost immediately, Jordan, von Neumann and Wigner showed [21] that all formally real Jordan algebras are direct sums of simple such algebras, with the latter falling into just five classes, parametrized by positive integers *n*: the self-adjoint parts, $M_{n}{\left(F\right)}_{\mathrm{sa}}$, of matrix algebras $M_{n}\left(F\right)$, where F=R,C or H (the quaternions) or, for n=3, over O (the octonions); and also what are called spin factors $V_{n}$ (closely related to Clifford algebras). There is some overlap: $V_{2}≃M_{2}\left(R\right),V_{3}≃M_{2}\left(C\right)$ and $V_{5}≃M_{2}\left(H\right)$. In all but one case, one can show that a simple Jordan algebra is a Jordan subalgebra of $M_{n}\left(C\right)$ for suitable *n*. The exceptional Jordan algebra, $M_{3}{\left(O\right)}_{\mathrm{sa}}$, admits no such representation.

Besides this classification theorem, there is only one other important fact about Euclidean Jordan algebras that is needed for what follows. This is the Koecher–Vinberg (KV) theorem alluded to above. Recall that an ordered vector space is a real vector space, call it E, spanned by a distinguished convex cone $E_{+}$ having its vertex at the origin. Such a cone induces a translation-invariant partial order on E, namely a≤b iff $b-a\in E_{+}$. As an example, the space $L_{\mathrm{sa}}\left(H\right)$ is ordered by the cone of positive operators. More generally, any EJA is an ordered vector space, with positive cone $E_{+}:=\left\{a^{2}|a\in A\right\}$. This cone has two special features: first, it is *homogeneous*, i.e., for any points a,b in the interior of $E_{+}$, there exists an automorphism of the cone (a linear isomorphism E→E, taking $E_{+}$ onto itself) that maps *a* to *b*. In other words, the group of automorphisms of the cone acts transitively on the cone’s interior. The other special property is that $E_{+}$ is *self-dual*. This means that E carries an inner product (in fact, the given one making E Euclidean) such that $a\in E_{+}$ iff 〈a,b〉≥0 for all $b\in E_{+}$.

An *order unit* in an ordered vector space E is an element $u\in E_{+}$ such that, for all a∈E, there exists some n∈N with a≤nu. In finite dimensions, this is equivalent to *u*’s belonging to the interior of the cone $E_{+}$ [22]. In the following, by a Euclidean order unit space, I mean an ordered vector space E equipped with an inner product 〈,〉 with 〈a,b〉≥0 for all $a,b\in E_{+}$, and a distinguished order-unit *u*. I will say that such a space E is HSD iff $E_{+}$ is homogeneous, and also self-dual with respect to the given inner product.

**Theorem** **1** (Koecher 1958; Vinberg 1961). Let E be a finite-dimensional euclidean order-unit space. If E is HSD, then there exists a unique product · with respect to which E (with its given inner product) is a euclidean Jordan algebra, u is the Jordan unit, and $E_{+}$ is the cone of squares.

It seems, then, that if we can motivate a representation of physical systems in terms of HSD order-unit spaces, we will have “reconstructed” what with a little license we might call finite-dimensional Jordan-quantum mechanics. In view of the classification theorem glossed above, this gets us into the neighborhood of orthodox QM, but still leaves open the possibility of taking real and quaternionic quantum systems seriously. (It also leaves the door open to two possibly unwanted guests, namely spin factors and the exceptional Jordan algebra. I will discuss below some constraints that at least bar the latter.)

**Some notational conventions.** My notation is mostly consistent with the following conventions (more standard in the mathematics than the physics literature, but in places slightly excentric relative to either). Capital Roman letters A,B,C serve as labels for systems. $M_{n}\left(F\right)$ stands for the set of n×n matrices over F=R or H; $M_{n}{\left(F\right)}_{\mathrm{sa}}$ is the set of self-adjoint such matrices. Vectors in a Hilbert space H are denoted by little Roman letters x,y,z from the end of the alphabet. Operators on H will usually be denoted by little Roman letters a,b,c,… from the beginning of the alphabet. Roman letters t,s typically stand for real numbers. The space of all linear operators on H is denoted L(H); as already indicated above, $L_{\mathrm{sa}}\left(H\right)$ is the (real) vector space of self-adjoint operators on H.

As above, the conjugate Hilbert space is denoted $\bar{H}$. I will write $\bar{x}$ for the vectors in $\bar{H}$ corresponding to x∈H. From a certain point of view, this is the same vector; the bar serves to remind us that $\bar{cx}=\bar{c}\;\bar{x}$ for scalars c∈C. Alternatively, one can regard $\bar{H}$ as the space of “bra” vectors 〈x| corresponding to the “kets” |x〉 in H, i.e., as the dual space of H.

The inner product of x,y∈H is written as 〈x,y〉 and is linear in the first argument (if you like: 〈x,y〉=〈y|x〉 in Dirac notation). The inner product on $\bar{H}$ is then $\langle \bar{x},\bar{y}\rangle =\langle y,x\rangle$. The rank-one projection operator associated with a unit vector x∈H is $p_{x}$. Thus, $p_{x}\left(y\right)=\langle y,x\rangle x$. I denote functionals on $L_{\mathrm{sa}}\left(H\right)$ by little Greek letters, e.g., α,β…, and operators on $L_{\mathrm{sa}}\left(H\right)$ by capital Greek letters, e.g., Φ. Two exceptions to this scheme: a generic density operator on H is denoted by the capital Roman letter *W*, and a certain special unit vector in $H⊗\bar{H}$ is denoted by the capital Greek letter Ψ. With luck, context will help keep things straight.

## 2. Homogeneity and Self-Duality in Quantum Theory

Why should a probabilistic physical system be represented by a Euclidean order-unit space that is either homogeneous or self-dual? One place to start hunting for an answer might be to look at standard quantum probability theory, to see if we can isolate, in operational or probabilistic terms, what makes this self-dual and homogeneous.

**Correlation and self-duality.** Let H be a finite-dimensional complex Hilbert space, representing some finite-dimensional quantum system. The system’s states are represented by density operators, i.e., positive trace-one operators $W\in L_{\mathrm{sa}}\left(H\right)$; possible measurement-outcomes are represented by effects, i.e., positive operators $a\in L_{\mathrm{sa}}\left(H\right)$ with a≤1. The Born rule specifies the probability of observing effect *a* in state *W* as Tr(Wa). If *W* is a pure state, i.e., $W=p_{v}$ where *v* is a unit vector in H, then Tr(Wa)=〈av,v〉; by the same token, if $a=p_{x}$, then Tr(Wa)=〈Wx,x〉.

For $a,b\in L_{\mathrm{sa}}\left(H\right)$, let 〈a,b〉:=Tr(ab). This is an inner product. By the spectral theorem, Tr(ab)≥0 for all $b\in L_{h}{\left(H\right)}_{+}$ iff $\mathrm{Tr}(ap_{x})\geq 0$ for all unit vectors *x*. However, $\mathrm{Tr}\left(ap_{x}\right)=\langle ax,x\rangle$. So Tr(ab)≥0 for all $b\in L_{h}{\left(H\right)}_{+}$ iff $a\in L_{h}{\left(H\right)}_{+}$, i.e., the trace inner product is self-dualizing. However, this now leaves us with the following:

**Question:** *What does the trace inner product* represent, *oprationally or probabilistically?*

Let $\bar{H}$ be the conjugate Hilbert space to H. Suppose H has dimension *n*. Any unit vector Ψ in $H⊗\bar{H}$ gives rise to a joint probability assignment to effects *a* on H and $\bar{b}$ on $\bar{H}$, namely $\langle \left(a⊗\bar{b}\right)\Psi ,\Psi \rangle$. Consider the EPR state for $H⊗\bar{H}$ defined by the unit vector:

$$\Psi =\frac{1}{\sqrt{n}}\sum_{x\in E}x⊗\bar{x}\in H⊗\bar{H},$$

where *E* is any orthonormal basis for H. A straightforward computation shows that the joint probability of observing *a* and *b* in the state Ψ is:

$$\langle \left(a⊗\bar{b}\right)\Psi ,\Psi \rangle =\frac{1}{n}\mathrm{Tr}\left(ab\right).$$

In other words, the normalized trace inner product just is the joint probability function determined by the pure state vector Ψ!

As a consequence, the state represented by Ψ has a very strong correlational property: if x,y are two orthogonal unit vectors with corresponding rank-one projections $p_{x}$ and $p_{y}$, we have $p_{x}p_{y}=0$, so $\langle \left(p_{x}⊗\bar{p_{y}}\right)\Psi ,\Psi \rangle =0$. On the other hand, $\langle \left(p_{x}⊗\bar{p_{x}}\right)\Psi ,\Psi \rangle =\frac{1}{n}\mathrm{Tr}\left(p_{x}\right)=\frac{1}{n}$. Hence, Ψ perfectly, and uniformly, correlates every basic measurement (orthonormal basis) of H with its counterpart in $\bar{H}$.

**Filters and homogeneity.** Next, let us see why the cone $L_{h}{\left(H\right)}_{+}$ is homogeneous. Recall that this means that any state in the interior of the cone (here, any non-singular density operator) can be obtained from any other by an automorphism of the cone. However, in fact, something better is true: this order-automorphism can be chosen to represent a probabilistically-reversible physical process, i.e., an invertible CP mapping with a CP inverse.

To see how this works, suppose *W* is a positive operator on H. Consider the pure CP mapping $\Phi_{W}:L_{\mathrm{sa}}\left(H\right)\to L_{\mathrm{sa}}\left(H\right)$ given by:

$$\Phi_{W}\left(a\right)=W^{1/2}aW^{1/2}.$$

Then, $\Phi_{W}\left(1\right)=W$. If *W* is nonsingular, so is $W^{1/2}$, so $\Phi_{W}$ is invertible, with inverse $\Phi_{W}^{-1}=\Phi_{W^{-1}}$, again a pure CP mapping. Now, given another nonsingular density operator *M*, we can get from *W* to *M* by applying $\Phi_{M}\circ \Phi_{W^{-1}}$.

All well and good, but we are still left with the following:

**Question:** *What does the mapping $\Phi_{W}$ represent, physically?*

To answer this, suppose *W* is a density operator, with spectral expansion $W=\sum_{x\in E}t_{x}p_{x}$. Here, *E* is an orthonormal basis for H diagonalizing *W*, and $t_{x}$ is the eigenvalue of *W* corresponding to x∈E. Then, for each vector x∈E,

$$\Phi_{W}\left(p_{x}\right)=t_{x}p_{x}$$

where $p_{x}$ is the projection operator associated with *x*. We can understand this to mean that $\Phi_{W}$ acts as a filter on the test *E*: the response of each outcome x∈E is attenuated by a factor $0\leq t_{x}\leq 1$ (my usage here is slightly non-standard, in that I allow filters that “pass” the system with a probability strictly between zero and one). Thus, if *M* is another density operator on H, representing some state of the corresponding system, then the probability of obtaining outcome *x* after preparing the system in state *M* and applying the process Φ is $t_{x}$ times the probability of *x* in state *M*. In detail: suppose $p_{x}$ is the rank-one projection operator associated with *x*, and note that $W^{1/2}p_{x}=p_{x}W^{1/2}=t_{x}^{1/2}p_{x}$. Thus,

$$\begin{array}{ccc} \mathrm{Tr}\left(\Phi_{W}\left(M\right)p_{x}\right)=\mathrm{Tr}\left(W^{1/2}MW^{1/2}p_{x}\right) & = & \mathrm{Tr}\left(W^{1/2}Mt_{x}^{1/2}p_{x}\right)=\mathrm{Tr}\left(t_{x}^{1/2}p_{x}W^{1/2}M\right)\\ & = & \mathrm{Tr}\left(t_{x}p_{x}M\right)=t_{x}\mathrm{Tr}\left(Mp_{x}\right). \end{array}$$

**Figure 1:**
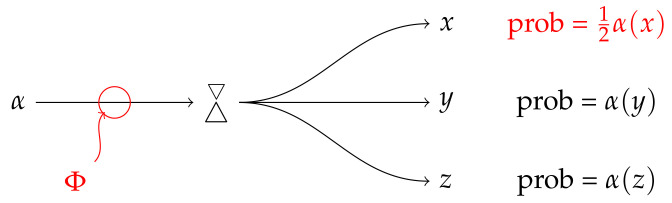
Φ attenuates *x*’s sensitivity by 1/2.

If we think of the basis *E* as representing a set of alternative channels plus detectors, as in the figure below, we can add a classical filter attenuating the response of one of the detectors (say, *x*) by a fraction $t_{x}$. What the computation above tells us is that we can achieve the same result by applying a suitable CP map to the system’s state. Moreover, this can be done independently for each outcome of *E*. In Figure 1, this is illustrated for a three-level quantum system: E={x,y,z} is an orthonormal basis, representing three possible outcomes of a Stern–Gerlach-like experiment; the filter Φ acts on the system’s state in such a way that the probability of outcome *x* is attenuated by a factor of $t_{x}=1/2$, while outcomes *y* and *z* are unaffected. Returning to the general situation, if we apply a filter $\Phi_{W}$ to the maximally-mixed state $\frac{1}{n}$, we obtain $\frac{1}{n}W$. Thus, we can prepare *W*, up to normalization, by applying the filter $\Phi_{W}$ to the maximally mixed state.

**Filters are symmetric.** Here is a final observation, linking these last two: the filter $\Phi_{W}$ is symmetric with respect to the uniformly-correlating “EPR” state Ψ, in the sense that:

$$\langle \left(\Phi_{W}\left(a\right)⊗\bar{b}\right)\Psi ,\Psi \rangle =\langle \left(a⊗{\bar{\Phi }}_{W}\left(b\right)\right)\Psi ,\Psi \rangle$$

for all effects $a,b\in L_{\mathrm{sa}}{\left(H\right)}_{+}$. Remarkably, this is all that is needed to recover the Jordan structure of finite-dimensional quantum theory: the existence of a conjugate system, with a uniformly-correlating joint state, plus the possibility of preparing non-singular states by means of filters that are symmetric with respect to this state, and doing so reversibly when the state is nonsingular.

In a very rough outline, the argument is that states preparable (up to normalization) by symmetric filters have spectral decompositions, and the existence of spectral decompositions makes the uniformly-correlating joint state a self-dualizing inner product. However, to spell this out in a precise way, I need a general mathematical framework for discussing states, effects and processes in abstraction from quantum theory. The next section reviews the necessary apparatus.

## 3. General Probabilistic Theories

A characteristic feature of quantum mechanics is the existence of incompatible, or non-comeasurable, observables. This suggests the following simple, but very fruitful, notion:

**Definition** **1.** A test space is a collection M of non-empty sets E,F,…, each representing the outcome-set of some measurement, experiment, or test. At the outset, one makes no special assumptions about the combinatorial structure of M. In particular, distinct tests are permitted to overlap. Let X:=⋃M denote the set of all outcomes of all tests in M: a probability weight on M is a function α:X→[0,1] such that $\sum_{x\in E}\alpha \left(x\right)=1$ for every E∈M.

Test spaces were introduced and studied by D. J. Foulis and C. H. Randall in a long series of papers beginning around 1970. The original term for a test was an operation, which has the advantage of signaling that the concept has wider applicability than simply reading a number off a meter: anything an agent can do that leads to a well-defined, exhaustive set of mutually-exclusive outcomes defines an operation. Accordingly, test spaces were originally called “manuals of operations”.

It can happen that a test space admits no probability weights at all. However, to serve as a model of a real family of experiments associated with an actual physical system, a test space should obviously carry a lavish supply of such weights. One might want to single out some of these as describing physically (or otherwise) possible states of the system. This suggests the following:

**Definition** **2.** A probabilistic model is a pair A=(M,Ω), where M is a test space and Ω is some designated convex set of probability weights, called the states of the model.

The definition is deliberately spare. Nothing prohibits us from adding further structure (a group of symmetries, say, or a topology on the space of outcomes). However, no such additional structure is needed for the results I will discuss below. I will write M(A),X(A) and Ω(A) for the test space, associated outcome space and state space of a model *A*. The convexity assumption on Ω(A) is intended to capture the possibility of forming mixtures of states. To allow the modest idealization of taking outcome-wise limits of states to be states, I will also assume that Ω(A) is closed as a subset of ${\left[0,1\right]}^{X(A)}$ (in its product topology). This makes Ω(A) compact and, so, guarantees the existence of pure states, that is, extreme points of Ω(A). If Ω(A) is the set of all probability weights on M(A), I will say that *A* has a full state space.

**Figure 2:**
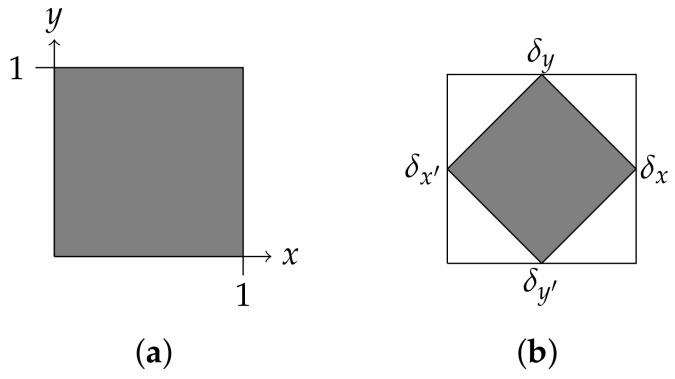
The state spaces of two bits. (**a**) The square bit; (**b**) The diamond bit.

**Two bits.** Here is a simple, but instructive illustration of these notions. Consider a test space $M=\{\left\{x,x^{\prime }\right\},\left\{y,y^{\prime }\right\}\}$. Here, we have two tests, each with two outcomes. We are permitted to perform either test, but not both at once. A probability weight is determined by the values it assigns to *x* and to *y*, and since the sets $\left\{x,x^{\prime }\right\}$ and $\left\{y,y^{\prime }\right\}$ are disjoint, these values are independent. Thus, geometrically, the space of all probability weights is the unit square in $R^{2}$ (Figure 2a, below). To construct a probabilistic model, we can choose any closed, convex subset of the square for Ω. For instance, we might let Ω be the convex hull of the four probability weights $\delta_{x},\delta_{x^{\prime }}$, $\delta_{y}$ and $\delta_{y^{\prime }}$ corresponding to the midpoints of the four sides of the square, as in Figure 2b, that is,

$$\delta_{x}\left(x\right)=1,\;\delta_{x}\left(x^{\prime }\right)=0,\;\delta_{x}\left(y\right)=\delta_{x}\left(y^{\prime }\right)=1/2,$$

$$\delta_{x^{\prime }}\left(x\right)=0,\;\delta_{x^{\prime }}\left(x^{\prime }\right)=1,\;\delta_{x^{\prime }}\left(y\right)=\delta_{x^{\prime }}\left(y^{\prime }\right)=1/2,$$

and similarly for $\delta_{y}$ and $\delta_{y^{\prime }}$.

The model of Figure 2a, in which we take Ω to be the entire set of probability weights on $M=\{\left\{x,x^{\prime }\right\},\left\{y,y^{\prime }\right\}\}$, is sometimes called the *square bit*. I will call the model of Figure 2b the *diamond bit*.

**Classical, quantum and Jordan models.** If *E* is a finite set, the corresponding *classical model* is A(E)=({E},Δ(E)) where Δ(E) is the simplex of probability weights on *E*. If H is a finite-dimensional complex Hilbert space, let M(H) denote the set of orthonormal bases of H: then X=⋃M(H) is the unit sphere of H, and any density operator *W* on H defines a probability weight $\alpha_{W}$, given by $\alpha_{W}\left(x\right)=\langle Wx,x\rangle$ for all x∈X. Letting Ω(H) denote the set of states of this form, we obtain the *quantum model*, A(H)=(M(H),Ω(H)), associated with H (Gleason’s theorem tells us that A(H) has a full state space for dim(H)>2, but we will not need this fact).

More generally, every Euclidean Jordan algebra E gives rise to a probabilistic model as follows. A minimal or primitive idempotent of E is an element p∈E with $p^{2}=p$ and, for $q=q^{2}<p$, q=0. A Jordan frame is a maximal pairwise orthogonal set of primitive idempotents. Let X(E) be the set of primitive idempotents; let M(E) be the set of Jordan frames; and let Ω(E) be the set of probability weights of the form α(p)=〈a,p〉 where $a\in E_{+}$ with 〈a,u〉=1. These data define the *Jordan model* A(E) associated with E. In the case where $E=L_{h}\left(H\right)$ for a finite-dimensional Hilbert space H, this almost gives us back the quantum model A(H): the difference is that we replace unit vectors by their associated projection operators, thus conflating outcomes that differ only by a phase.

**Sharp models.** Jordan models enjoy many special features that the generic probabilistic model lacks. I want to take a moment to discuss one such feature, which will be important below.

**Definition** **3.** A model A is unital iff, for every outcome x∈X(A), there exists a state α∈Ω(A) with α(x)=1, and sharp if this state is unique (from which it follows easily that it must be pure). If A is sharp, I will write $\delta_{x}$ for the unique state making x∈X(A) certain.

If *A* is sharp, then there is a sense in which each test E∈M(A) is maximally informative: if we are certain which outcome x∈E will occur, then we know the system’s state exactly, as there is only one state in which *x* has probability 1.

Classical and quantum models are obviously sharp. More generally, every Jordan model is sharp. To see this, note first that every state α on a Euclidean Jordan algebra E has the form α(x)=〈a,x〉 where $a\in E_{+}$ with 〈a,u〉=1 and where 〈,〉 is the given inner product on E, normalized so that ∥x∥=1 for all primitive idempotents (equivalently, so that ∥u∥=n, the rank of E). The spectral theorem for EJAs [13] shows that $a=\sum_{p\in E}t_{p}p$ where *E* is a Jordan frame and the coefficients $t_{p}$ are non-negative and sum to one (since 〈a,u〉=1). If 〈a,x〉=1, then $\sum_{p\in E}t_{p}\langle p,x\rangle =1$ implies that, for every p∈E with $t_{p}>0$, 〈p,x〉=1. However, ∥p∥=∥x∥=1, so this implies that 〈p,x〉=∥p∥∥x∥, which in turn implies that p=x.

In general, a probabilistic model need not even be unital, much less sharp. On the other hand, given a unital model *A*, it is often possible to construct a sharp model by suitably restricting the state space. This is illustrated in Figure 2b above: the full state space of the square bit is unital, but far from sharp; however, by restricting the state space to the convex hull of the barycenters of the faces, we obtain a sharp model. This is possible whenever *A* is unital and carries a group of symmetries acting transitively on the outcome-set X(A). For details, see Appendix A. The point here is that sharpness is not, by itself, a very stringent condition: since we should expect to find highly symmetric, unital models represented abundantly “in nature”, we can also expect to encounter an abundance of systems represented by sharp models.

The spaces V(A), $V^{*}\left(A\right)$. Any probabilistic model gives rise to a pair of ordered vector spaces in a canonical way. These will be essential in the development below, so I am going to go into a bit of detail here.

**Definition** **4.** *Let A be any probabilistic model. Let V(A) be the span of the state space Ω(A) in $R^{X(A)}$, ordered by the cone $V{\left(A\right)}_{+}$ consisting of non-negative multiples of states, i.e.,*

$$V{\left(A\right)}_{+}=\left\{t\alpha |\alpha \in \Omega \left(A\right),\;t\geq 0\right\}.$$

Call the model *A* finite-dimensional iff V(A) is finite-dimensional. From now on, I assume that all models are finite-dimensional.

Let $V^{*}\left(A\right)$ denote the dual space of V(A), ordered by the dual cone of positive linear functionals, i.e., functionals *f* with f(α)≥0 for all $\alpha \in V{\left(A\right)}_{+}$. Any measurement-outcome x∈X(A) yields an evaluation functional $\hat{x}\in V^{*}\left(A\right)$, given by $\hat{x}\left(\alpha \right)=\alpha \left(x\right)$ for all α∈V(A). More generally, an effect is a positive linear functional $f\in V^{*}\left(A\right)$ with 0≤f(α)≤1 for every state α∈Ω(A). The functionals $\hat{x}$ are effects. One can understand an arbitrary effect *a* to represent a mathematically possible measurement outcome, having probability a(α) in state α. I stress the adjective mathematically because, a priori, there is no guarantee that every effect will correspond to a physically-realizable measurement outcome. In fact, at this stage, I make no assumption at all about what, apart from the tests E∈M(A), is or is not physically realizable. (Later, it will follow from further assumptions that every element of $V^{*}\left(A\right)$ represents a random variable associated with some E∈M(A) and is, therefore, operationally meaningful. However, this will be a theorem, not an assumption.)

The *unit effect* is the functional $u_{A}:=\sum_{x\in E}\hat{x}$, where *E* is any element of M(A). This takes the constant value of one on Ω(A), and, thus, represents a trivial measurement outcome that occurs with probability one in every state. This is an order unit for $V^{*}\left(A\right)$ (to see this, let $a\in V{\left(A\right)}^{*}$, and let *N* be the maximum value of |a(α)| for α∈Ω(A), remembering that the latter is compact: then a≤Nu).

For both classical and quantum models, the ordered vector spaces $V^{*}\left(A\right)$ and V(A) are naturally isomorphic. If A(E) is the classical model associated with a finite set *E*, both are isomorphic to the space $R^{E}$ of all real-valued functions on *E*, ordered pointwise. If A=A(H) is the quantum model associated with a finite-dimensional Hilbert space H, V(A) and $V^{*}\left(A\right)$ are both naturally isomorphic to the space $L_{h}\left(H\right)$ of Hermitian operators on H, ordered by its usual cone of positive semi-definite operators. More generally, if E is a Euclidean Jordan algebra and A=A(E) is the corresponding Jordan model, then $V\left(A\right)≃E≃V^{*}\left(A\right)$, with E ordered as usual, i.e., by its cone of squares. The first of these isomorphisms is due to the definition of the model A(E) and the second to E’s self-duality.

**The space E(A).** It is going to be technically useful to introduce a third ordered vector space, which I will denote by E(A). This is the span of the evaluation-effects $\hat{x}$, associated with measurement outcomes x∈X(A), in $V^{*}\left(A\right)$, ordered by the cone:

$$E{\left(A\right)}_{+}\;:=\;\left\{\;\sum_{i}t_{i}{\hat{x}}_{i}\;|\;t_{i}\geq 0\;\right\}.$$

**Figure 3:**
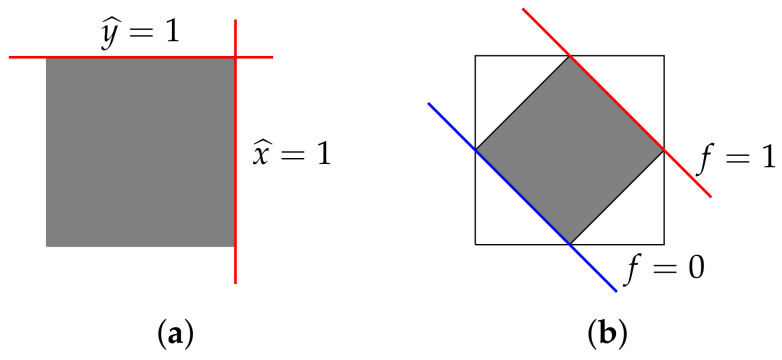
(**a**) Two outcome-effects for the square bit; (**b**) An effect for the diamond bit not positive on the square bit.

That is, $E{\left(A\right)}_{+}$ is the set of linear combinations of effects $\hat{x}$ having non-negative coefficients. It is important to note that this is, in general, a proper sub-cone of $V{\left(A\right)}_{+}^{*}$. To see this, we can revisit the example of the “diamond bit” of Figure 2b. Letting *x* and *y* be the outcomes corresponding to the right face and the top face of the larger (full) state space pictured below in Figure 3a, consider the functional $f:=\hat{x}+\hat{y}-\frac{1}{2}u$. This takes positive values on the smaller state space of the diamond bit, but is negative on, for example, the state γ corresponding to the lower-left corner of the full state space (see Figure 3b). Thus, $f\in V{\left(A\right)}_{+}$, but $f\notin E{\left(A\right)}_{+}$.

Since we are working in finite dimensions, the outcome-effects $\hat{x}$ span $V^{*}\left(A\right)$. Thus, as vector spaces, E(A) and $V^{*}\left(A\right)$ are the same. However, as the diamond bit illustrates, they can have quite different positive cones and, thus, need not be isomorphic as ordered vector spaces.

**Processes and subnormalized states.** A *subnormalized state* of a model *A* is an element α of $V{\left(A\right)}_{+}$ with u(α)<1. These can be understood as states that allow a nonzero probability 1−u(α) of some generic “failure” event, (e.g., the destruction of the system), represented by the zero functional in $V^{*}\left(A\right)$.

More generally, we may wish to regard two systems, represented by models *A* and *B*, as the input to and output from some process, whether dynamical or purely information-theoretic, that has some probability to destroy the system or otherwise “fail”. Since such a process should preserve probabilistic mixtures, it should be represented mathematically by an affine mapping $T:\Omega \left(A\right)\to V{\left(B\right)}_{+}$, taking each normalized state α of *A* to a possibly sub-normalized state T(α) of *B*. One can show that such a mapping extends uniquely to a positive linear mapping:

T:V(A)→V(B),

so from now on, this is how I represent processes.

Even if a process *T* has a nonzero probability of failure, it may be possible to reverse its effect with nonzero probability.

**Definition** **5.** A process T:A→B is probabilistically reversible iff there exists a process S such that, for all α∈Ω(A), (S∘T)(α)=pα, where p∈(0,1].

This means that there is a probability 1−p of the composite process S∘T failing, but a probability *p* that it will leave the system in its initial state (note that, since S∘T is linear, *p* must be constant); where *T* preserves normalization, so that T(Ω(A))⊆Ω(B), *S* can also be taken to be normalization-preserving and will undo the result of *T* with probability one. This is the more usual meaning of “reversible” in the literature.

Given a process T:V(A)→V(B), there is a dual mapping $T^{*}:V^{*}\left(B\right)\to V^{*}\left(A\right)$, also positive, given by $T^{*}\left(b\right)\left(\alpha \right)=b\left(T\left(\alpha \right)\right)$ for all $b\in V^{*}\left(B\right)$ and α∈V(A). The assumption that *T* takes normalized states to subnormalized states is equivalent to the requirement that $T^{*}\left(u_{B}\right)\leq u_{A}$, that is that $T^{*}$ maps effects to effects.

**Remark** **1.** Since we are attaching no special physical interpretation to the cone $E_{+}\left(A\right)$, we do not require a physical process T:V(A)→V(B) to have a dual process $T^{*}$ that maps $E_{+}\left(B\right)$ to $E_{+}\left(A\right)$. That is, we do not require $T^{*}$ to be positive as a mapping E(B)→E(A).

**Joint probabilities and joint states.** If $M_{1}$ and $M_{2}$ are two test spaces, with outcome-spaces $X_{1}$ and $X_{2}$, we can construct a space of product tests (note here the savage abuse of notation: $M_{1}\times M_{2}$ is not the Cartesian product of $M_{1}$ and $M_{2}$):

$$M_{1}\times M_{2}=\left\{\;E\times F\;|\;E\in M_{1},F\in M_{2}\;\right\}$$

This models a situation in which tests from $M_{1}$ and from $M_{2}$ can be performed separately, and the results collated. Note that the outcome-space for $M_{1}\times M_{2}$ is $X_{1}\times X_{2}$. A joint probability weight on $M_{1}$ and $M_{2}$ is just a probability weight on $M_{1}\times M_{2}$, that is a function $\omega :X_{1}\times X_{2}\to \left[0,1\right]$ such that $\sum_{(x,y)\in E\times F}\omega \left(x,y\right)=1$ for all tests $E\in M_{1}$ and $F\in M_{2}$. One says that ω is non-signaling iff the marginal (or reduced) probability weights $\omega_{1}$ and $\omega_{2}$, given by:

$$\omega_{1}\left(x\right)=\sum_{y\in F}\omega \left(x,y\right)\;\;\mathrm{and}\;\;\omega_{2}\left(y\right)=\sum_{x\in E}\omega \left(x,y\right)$$

are well-defined, i.e., independent of the choice of the tests *E* and *F*, respectively. One can understand this to mean that the choice of which test to measure on $M_{1}$ has no observable, i.e., no statistical, influence on the outcome of tests made of $M_{2}$, and vice versa. In this case, one also has well-defined conditional probability weights:

$$\omega_{2|x}\left(y\right):=\omega \left(x,y\right)/\omega_{1}\left(x\right)\;\;\mathrm{and}\;\;\omega_{1|y}:=\omega \left(x,y\right)/\omega_{2}\left(y\right)$$

(with, say, $\omega_{2|x}=0$ if $\omega_{1}\left(x\right)=0$, and similarly for $\omega_{1|y}$). This gives us the following bipartite version of the law of total probability [23]: for any choice: of $E\in M_{1}$ or $F\in M_{2}$,

(1) $$\omega_{2}=\sum_{x\in E}\omega_{1}\left(x\right)\omega_{2|x}\;\;\;\mathrm{and}\;\;\omega_{1}=\sum_{y\in F}\omega_{2}\left(y\right)\omega_{1|y}.$$

**Definition** **6.** A joint state on a pair of probabilistic models A and B is a non-signaling joint probability weight ω on M(A)×M(B) such that, for every x∈X(A) and every y∈X(B), the conditional probability weights $\omega_{2|x}$ and $\omega_{1|y}$ belong to Ω(A) and Ω(B), respectively. It follows from (1) that the marginal weights $\omega_{1}$ and $\omega_{2}$ are also states of A and B, respectively.

This naturally suggests that one should define, for models *A* and *B*, a composite model AB, the states of which would be precisely the joint states on *A* and *B*. If one takes M(AB)=M(A)×M(B), this is essentially the “maximal tensor product” of *A* and *B* [24]. However, this does not coincide with the usual composite of quantum-mechanical systems. In Section 6, I will discuss composite systems in more detail. Meanwhile, for the main results of this paper, the idea of a joint state is sufficient.

For a simple example of a joint state that is neither classical, nor quantum, let *B* denote the “square bit” model discussed above. That is, B=(B,Ω) where e $B=\{\left\{x,x^{\prime }\right\},\left\{y,y^{\prime }\right\}\}$ is a test space with two non-overlapping, two-outcome tests, and Ω is the set of all probability weights thereon, amounting to the unit square in $R^{2}$. The joint state on B×B given by Table 1 (a variant of the “non-signaling box” of Popescu and Rohrlich [25]) is clearly non-signaling. Notice that it also establishes a perfect, uniform correlation between the outcomes of any test on the first system and its counterpart on the second.

**Conditioning maps.** If ω is a joint state on *A* and *B*, define the associated *conditioning maps* $\;\hat{\omega }:X\left(A\right)\to V\left(B\right)\;\;\mathrm{and}\;\;{\hat{\omega }}^{*}:X\left(B\right)\to V\left(A\right)$ by:

$$\hat{\omega }\left(x\right)\left(y\right)\;=\;\omega \left(x,y\right)\;=\;{\hat{\omega }}^{*}\left(y\right)\left(x\right)$$

for all x∈X(A) and y∈X(B). Note that $\hat{\omega }\left(x\right)=\omega_{1}\left(x\right)\omega_{2|x}$ for every x∈X(A), i.e., $\hat{\omega }\left(x\right)$ can be understood as the un-normalized conditional state of *B* given the outcome *x* on *A*. Similarly, ${\hat{\omega }}^{*}\left(y\right)$ is the unnormalized conditional state of *A* given outcome *y* on *B*.

The conditioning map $\hat{\omega }$ extends uniquely to a positive linear mapping E(A)→V(B), which I also denote by $\hat{\omega }$, such that $\hat{\omega }\left(\hat{x}\right)=\hat{\omega }\left(x\right)$ for all outcomes x∈X(A). To see this, consider the linear mapping $T:V^{*}\left(A\right)\to R^{X(B)}$ defined, for $f\in V^{*}\left(A\right)$, by $T\left(f\right)\left(y\right)=f({\hat{\omega }}^{*}\left(y\right))$ for all y∈X(B). If $f=\hat{x}$, we have $T\left(\hat{x}\right)=\omega_{1}\left(x\right)\omega_{2|x}\in V{\left(B\right)}_{+}$, whence, for all y∈X(B), $T\left(\hat{x}\right)\left(y\right)=\omega \left(x,y\right)=\hat{\omega }\left(x\right)\left(y\right)$. Since the evaluation functionals $\hat{x}$ span E(A), the range of *T* lies in V(B), and moreover, *T* is positive on the cone $E{\left(A\right)}_{+}$. Hence, as advertised, *T* defines a positive linear mapping E(B)→V(A), extending $\hat{\omega }$. In the same way, ${\hat{\omega }}^{*}$ defines a positive linear mapping ${\hat{\omega }}^{*}:E\left(B\right)\to V\left(A\right)$.

An immediate and important corollary is that any joint state ω on *A* and *B* defines a bilinear form, which by abuse of notation I also call ω, on E(A)×E(B), given by $\omega \left(a,b\right)\;:=\;\hat{\omega }\left(a\right)\left(b\right)$ for all a,b∈E(A). Note that $\omega \left(\hat{x},\hat{y}\right)=\omega \left(x,y\right)$ for all x∈X(A),y∈X(B) and also that the bilinear form ω is positive, in the sense that ω(a,b)≥0 for all $a\in E{\left(A\right)}_{+}$ and all $b\in E{\left(B\right)}_{+}$.

## 4. Conjugates and Filters

We are now in a position to abstract the two features of QM discussed earlier. Call a test space (X,M)*uniform* iff all tests E∈M have the same size, which we then call the *rank* of the test space. The test spaces associated with quantum models are uniform, and it is quite easy to generate many other examples (see Appendix A).

A uniform test space of rank *n* always admits at least one probability weight, namely the maximally-mixed probability weight ρ(x)=1/n for all x∈X. I will say that a probabilistic model *A* is uniform if the test space M(A) is uniform and the maximally-mixed state ρ belongs to Ω(A).

By an *isomorphism* γ:A→B from a probabilistic model *A* to a probabilistic model *B*, I mean the obvious thing: a bijection γ:X(A)→X(B) taking M(A) onto $M(\bar{A})$, and such that β↦β∘γ maps $\Omega (\bar{A})$ onto Ω(A).

**Definition** **7.** *Let A be uniform probabilistic model with tests of size n. A conjugate for A is a model $\bar{A}$, plus a chosen isomorphism $\gamma_{A}:A≃\bar{A}$ and a joint state $\eta_{A}$ on A and $\bar{A}$ such that for all x,y∈X(A),*

*(a)*  $\eta_{A}\left(x,\bar{x}\right)=1/n$
*(b)*  $\eta_{A}\left(x,\bar{y}\right)=\eta \left(y,\bar{x}\right)$

*where $\bar{x}:=\gamma_{A}\left(x\right)$.*

This corresponds to what is called a “weak conjugate” in [17]. Note that if E∈M(A), we have $\sum_{x,y\in E\times E}\eta_{A}\left(x,\bar{y}\right)=1$ and |E|=n. Hence, $\eta_{A}\left(x,\bar{y}\right)=0$ for x,y∈E with x≠y. Thus, $\eta_{A}$ establishes a perfect, uniform correlation between any test E∈M(A) and its counterpart, $\bar{E}:=\left\{\bar{x}|x\in E\right\}$, in $M(\bar{A})$.

The symmetry condition (b) is pretty harmless. If η is a joint state on *A* and $\bar{A}$ satisfying (a), then so is $\eta^{t}\left(x,\bar{y}\right):=\eta \left(y,\bar{x}\right)$; thus, $\frac{1}{2}\left(\eta +\eta^{t}\right)$ satisfies both (a) and (b). In fact, if *A* is sharp, (b) is automatic: if η satisfies (a), then the conditional state ${\left(\eta_{A}\right)}_{1|\bar{x}}$ assigns probability one to the outcome *x*. If *A* is sharp, this implies that $\eta_{1|\bar{x}}=\delta_{x}$ is uniquely defined, whence $\eta \left(x,\bar{y}\right)=n\delta_{y}\left(x\right)$ is also uniquely defined. In other words, for a sharp model *A* and a given isomorphism $\gamma :A≃\bar{A}$, there exists at most one joint state η satisfying (a); whence, in particular, $\eta =\eta^{t}$.

If A=A(H) is the quantum-mechanical model associated with an *n*-dimensional Hilbert space H, then we can take $\bar{A}=A\left(\bar{H}\right)$ and define $\eta_{A}\left(x,\bar{y}\right)={\left|\langle \Psi ,x⊗\bar{y}\rangle \right|}^{2}$, where Ψ is the EPR state on $H⊗\bar{H}$, as discussed in Section 3.

So much for conjugates. We generalize the filters associated with pure CP mappings as follows:

**Definition** **8.** A filter associated with a test E∈M(A) is a positive linear mapping Φ:V(A)→V(A) such that for every outcome x∈E, there is some coefficient $t_{x}\in \left[0,1\right]$ with $\Phi \left(\alpha \right)\left(x\right)=t_{x}\alpha \left(x\right)$ for every state α∈Ω(A).

Equivalently, Φ is a filter iff the dual process $\Phi^{*}:V^{*}\left(A\right)\to V^{*}\left(A\right)$ satisfies $\Phi^{*}\left(\hat{x}\right)=t_{x}\hat{x}$ for each x∈E. Just as in the quantum-mechanical case, a filter independently attenuates the “sensitivity” of the outcomes x∈E. (The extreme case is one in which the coefficient $t_{x}$ corresponding to a particular outcome is one, and the other coefficients are all zero. In that case, all outcomes other than *x* are, so to say, blocked by the filter. Conversely, given such an “all or nothing” filter $\Phi_{x}$ for each x∈E, we can construct an arbitrary filter with coefficients $t_{x}$ by setting $\Phi =\sum_{x\in E}t_{x}\Phi_{x}$.)

Call a filter Φ*reversible* iff Φ is an order-automorphism of V(A); that is, iff it is probabilistically reversible as a process. Evidently, this requires that all the coefficients $t_{x}$ be nonzero. We will eventually see that the existence of a conjugate, plus the preparability of arbitrary nonsingular states by symmetric reversible filters, will be enough to force *A* to be a Jordan model. Most of the work is done by the easy Lemma 1, below. First, some terminology.

**Definition** **9.** *Suppose $\Delta =\{\delta_{x}|x\in X\left(A\right)\}$ is a family of states indexed by outcomes x∈X(A) and such that $\delta_{x}\left(x\right)=1$. Say that a state α is spectral with respect to* Δ *iff there exists a test E∈M(A) such that $\alpha =\sum_{x\in E}\alpha \left(x\right)\delta_{x}$. Say that the model A itself is spectral with respect to* Δ *if every state of A is spectral with respect to* Δ.

If *A* has a conjugate $\bar{A}$, then the bijection $\gamma_{A}:X\left(A\right)\to X\left(\bar{A}\right)$ extends to an order-isomorphism $E\left(A\right)≃E\left(\bar{A}\right)$. It follows that every non-signaling joint probability weight ω on *A* and $\bar{A}$ defines a bilinear form $a,b\mapsto \omega (a,\bar{b})$ on E(A).

The following is essentially proven in [17], but the presentation here is somewhat different.

**Lemma** **1.** *Let A have a conjugate $\left(\bar{A},\eta_{A}\right)$. Suppose A is spectral with respect to the states $\delta_{x}:=\eta_{1|\bar{x}}$, x∈X(A). Then:*

$$\langle a,b\rangle :=n\eta_{A}\left(a,\bar{b}\right),$$

where n is the rank of A, defines a self-dualizing inner product on E(A), with respect to which $V{\left(A\right)}_{+}≃E{\left(A\right)}_{+}$. Moreover, A is sharp, and $E{\left(A\right)}_{+}=V^{*}{\left(A\right)}_{+}$.

**Proof.**  That 〈,〉 is symmetric and bilinear follows from $\eta_{A}$’s being symmetric and non-signaling. Note that $\langle \hat{x},\hat{x}\rangle =1$ for every x∈X(A) and $\langle \hat{x},\hat{y}\rangle =0$ for any distinct x,y∈X(A) lying in a common test. We need to show that 〈,〉 is positive-definite. Since $\hat{A}≃A$ and the latter is spectral, so is the former. It follows that $\hat{\eta }$ takes $E{\left(A\right)}_{+}$ onto $V{\left(\bar{A}\right)}_{+}$ and, hence, is an order-isomorphism. From this, it follows that every $a\in E{\left(A\right)}_{+}$ has a “spectral” decomposition of the form $\sum_{x\in E}t_{x}x$ for some coefficients $t_{x}\geq 0$ and some test E∈M(A). In fact, any a∈E(A), positive or otherwise, has such a decomposition (albeit with possibly negative coefficients). If a∈E(A) is arbitrary, with $a=a_{1}-a_{2}$ for some $a_{1},a_{2}\in E{\left(A\right)}_{+}$, we can find N≥0 with $a_{2}\leq Nu$. Thus, $b:=a+Nu=a_{1}+\left(Nu-a_{2}\right)\geq 0$, and so, $b:=\sum_{x\in E}t_{x}x$ for some E∈A, and hence, $a=b-Nu=\sum_{x\in E}t_{x}x-N\left(\sum_{x\in E}x\right)=\sum_{x\in E}\left(t_{x}-N\right)x$.

Now, let a∈E(A). Decomposing $a=\sum_{x\in E}t_{x}x$ for some test *E* and some coefficients $t_{x}$, we have:

$$\langle a,a\rangle =\sum_{x,y\in E\times E}t_{x}t_{y}\langle \hat{x},\hat{y}\rangle =\sum_{x\in E}{t_{x}}^{2}\geq 0.$$

This is zero only where all coefficients $t_{x}$ are zero, i.e., only for a=0. Therefore, 〈,〉 is an inner product, as claimed.

We need to show that 〈,〉 is self-dualizing. Clearly $\langle a,b\rangle =n\eta_{A}\left(a,\bar{b}\right)\geq 0$ for all $a,b\in E{\left(A\right)}_{+}$. Suppose a∈E(A) is such that 〈a,b〉≥0 for all $b\in E{\left(A\right)}_{+}$. Then, $\langle a,\hat{y}\rangle \geq 0$ for all y∈X. Now, $a=\sum_{x\in E}t_{x}\hat{x}$ for some test *E*; thus, for all y∈E, we have $\langle a,\hat{y}\rangle =t_{y}\geq 0$, whence, $a\in E{\left(A\right)}_{+}$.

Next, we want to show that $E{\left(A\right)}_{+}=V{\left(A\right)}_{+}^{*}$. Since $\hat{\eta }:E\left(A\right)\to V\left(\bar{A}\right)$ is an order-isomorphism, for every α∈V(A), there exists a unique a∈E(A) with $\hat{\eta }\left(a\right)=\frac{1}{n}\bar{\alpha }$. In particular,

$$\langle a,x\rangle =n\eta_{A}\left(a,\bar{x}\right)=\bar{\alpha }\left(\bar{x}\right)=\alpha \left(x\right).$$

It follows that if $b\in E\left(A\right)=V^{*}\left(A\right)$,

$$b\left(\alpha \right)=\bar{b}\left(\bar{\alpha }\right)=\bar{b}n{\hat{\eta }}_{A}\left(a\right)=n\eta \left(a,\bar{b}\right)=\langle a,b\rangle .$$

Since every $a\in E{\left(A\right)}_{+}$ has the form $a={\hat{\eta }}^{-1}\left(\frac{1}{n}\bar{\alpha }\right)$ for some $\alpha \in V{\left(A\right)}_{+}$, if $b\in V^{*}{\left(A\right)}_{+}$, we have 〈a,b〉≥0 for all $a\in E{\left(A\right)}_{+}$, whence, by the self-duality of the latter cone, $b\in E{\left(A\right)}_{+}$. Thus, $V^{*}\left(A\right)=E{\left(A\right)}_{+}$.

Finally, let us see that *A* is sharp. If α∈Ω(A), let *a* be the unique element of $E{\left(A\right)}_{+}$ with 〈a,x〉=α(x). In particular, 〈a,u〉=1. If *a* has spectral decomposition $a=\sum_{x\in E}t_{x}\hat{x}$, where E∈M(A), then for all x∈E, $\langle a,x\rangle =t_{x}$; hence, $\sum_{x\in E}t_{x}=\sum_{x\in E}\langle a,x\rangle =\langle a,u\rangle =1$. Thus, ${∥a∥}^{2}=\sum_{x\in E}t_{x}^{2}\leq 1$, whence, ∥a∥≤1. Now, suppose α(x)=1 for some x∈X(A): then, 1=〈a,x〉≤∥a∥∥x∥; as ∥x∥=1, we have ∥a∥=1. However, now $\langle a,\hat{x}\rangle =∥a∥∥\hat{x}∥$, whence, $a=\hat{x}$. Hence, there is only one weight α with α(x)=1, namely, α=〈x,·〉, so *A* is sharp. ☐

If *A* is sharp, then we say that *A* is *spectral* iff it is spectral with respect to the pure states $\delta_{x}$ defined by $\delta_{x}\left(x\right)=1$. If *A* is sharp and has a conjugate $\bar{A}$, then, as noted earlier, the state $\eta_{1|\bar{x}}$ is exactly $\delta_{x}$, so the spectrality assumption in Lemma 1 is fulfilled if we simply say that *A* is spectral. Hence, a sharp, spectral model with a conjugate is self-dual.

For the simplest systems, this is already enough to secure the desired representation in terms of a Euclidean Jordan algebra.

**Definition** **10.** Call A a bit iff it has rank two (that is, all tests have two outcomes) and if every state α∈Ω(A) can be expressed as a mixture of two sharply distinguishable states; that is, $\alpha =t\delta_{x}+\left(1-t\right)\delta_{y}$ for some t∈[0,1] and states $\delta_{x}$ and $\delta_{y}$ with $\delta_{x}\left(x\right)=1$ and $\delta_{y}\left(y\right)=1$ for some test {x,y}.

**Corollary** **1.** If A is a sharp bit, then Ω(A) is a ball of some finite dimension d.

The proof is given in Appendix C. If *d* is 2, 3 or 5, we have a real, complex or quaternionic bit. For d=4 or d≥6, we have a non-quantum spin factor.

For systems of higher rank (higher “information capacity”), we need to assume a bit more. Suppose *A* satisfies the hypotheses of Lemma 1. Appealing to the Koecher–Vinberg theorem, we see that if V(A) and, hence, $V^{*}\left(A\right)$ are also homogeneous, then $V^{*}\left(A\right)$ carries a canonical Jordan structure. In fact, we can say something a little stronger.

**Theorem** **2.** Let A be spectral with respect to a conjugate system $\bar{A}$. If V(A) is homogeneous, then there exists a canonical Jordan product on E(A) with respect to which $u_{A}$ is the Jordan unit. Moreover, with respect to this product, X(A) is exactly the set of primitive idempotents, and M(A) is exactly the set of Jordan frames.

The first part is almost immediate from the Koecher–Vinberg theorem, together with Lemma 1. The KV theorem gives us an isomorphism between the ordered vector spaces V(A) and E(A), so if one is homogeneous, so is the other. Since E(A) is also self-dual by Lemma 1, the KV theorem yields the requisite unique Euclidean Jordan structure having *u* as the Jordan unit. One can then show without much trouble that every outcome x∈X(A) is a primitive idempotent of E(A) with respect to this Jordan structure and that every test is a Jordan frame. The remaining claims (that every minimal idempotent belongs to X(A) and every Jordan frame, to M(A)) take a little bit more work. I will not reproduce the proof here; the details (which are not especially difficult, but depend on some facts concerning Euclidean Jordan algebras) can be found in [17].

The homogeneity of V(A) can be understood as a preparability assumption: it is equivalent to saying that every state in the interior of Ω(A) can be obtained, up to normalization, from the maximally-mixed state by a reversible process. That is, if α∈Ω(A), there is some such process ϕ such that ϕ(ρ)=pα where 0<p≤1. One can think of the coefficient *p* as the probability that the process ϕ will yield a nonzero result (more dramatically: will not destroy the system). Thus, if we prepare an ensemble of identical copies of the system in the maximally-mixed state ρ and subject them all to the process ϕ, the fraction that survives will be about *p*, and these will all be in state α.

In fact, if the hypotheses of Lemma 1 hold, the homogeneity of E(A) follows directly from the mere existence of reversible filters with arbitrary non-zero coefficients. To see this, suppose $a\in E{\left(A\right)}_{+}$ has a spectral decomposition $\sum_{x\in E}t_{x}\hat{x}$ for some E∈M(A), with $t_{x}>0$ for all *x* when *a* belongs to the interior of $E{\left(A\right)}_{+}$. Now, if we can find a reversible filter for *E* with $\Phi \left(x\right)=t_{x}\hat{x}$ for all x∈E, then applying this to the order-unit $u=\sum_{x\in E}\hat{x}$ yields *a*. Thus, $V^{*}\left(A\right)$ is homogeneous.

**Two paths to spectrality.** Some axiomatic treatments of quantum theory have taken one or another form of spectrality as an axiom [6,26]. If one is content to do this, then Lemma 1 above provides a very direct route to the Jordan structure of quantum theory. However, spectrality can actually be derived from assumptions that, on their face, seem a good deal weaker, or anyway more transparent (a different path to spectrality is charted in a recent paper [27] by G. Chiribella and C. M. Scandolo).

I will call a joint state on models *A* and *B* *correlating* iff it sets up a perfect correlation between some pair of tests E∈M(A) and F∈M(B). More exactly:

**Definition** **11.** A joint state ω on probabilistic models A and B correlates a test E∈M(A) with a test F∈M(B) iff there exist subsets $E_{0}\subseteq E$ and $F_{0}\subseteq F$, and a bijection $f:E_{0}\to F_{0}$ such that ω(x,y)=0 for (x,y)∈E×F unless y=f(x). In this case, say that ω correlates E with F along f. A joint state on A and B is correlating iff it correlates some pair of tests E∈M(A),F∈M(B).

Note that ω correlates *E* with *F* along *f* iff $\omega \left(x,f\left(x\right)\right)=\omega_{1}\left(x\right)=\omega_{2}\left(f\left(x\right)\right)$, which, in turn, is equivalent to saying that $\omega_{2|x}\left(f\left(x\right)\right)=1$ for $\omega_{1}\left(x\right)\neq 0$.

**Lemma** **2.** Suppose A is sharp and that every state α of A arises as the marginal of a correlating joint state between A and some model B. Then, A is spectral.

**Proof.**  Suppose $\alpha =\omega_{1}$, where ω is a joint state correlating a test E∈M(A) with a test F∈M(B), say along a bijection $f:E_{0}\to F_{0}$, where $E_{o}\subseteq E$ and $F_{0}\subseteq F$. Then, for any x∈E with α(x)≠0, $\omega_{1|f(x)}\left(x\right)=1$, whence, as *A* is sharp, $\omega_{1|f(x)}=\delta_{x}$, the unique state making *x* certain. It follows from the law of total probability that $\alpha =\sum_{x\in E}\alpha \left(x\right)\delta_{x}$. ☐

In principle, the model *B* can vary with the state α. Lemma 2 suggests the following language:

**Definition** **12.** A model A satisfies the correlation condition iff every state α∈Ω(A) is the marginal of some correlating joint state of A and some model B.

This has something of the same flavor as the *purification postulate* of [8], which requires that all states of a given system arise as marginals of a pure state on a larger, composite system, unique up to symmetries on the purifying system. However, note that we do not require the correlating joint state to be either pure (which, in classical probability theory, it will not be) or unique.

If *A* is sharp and satisfies the correlation condition, then every state of *A* is spectral. If, in addition, *A* has a conjugate, then for every x∈X(A), we have $\eta_{1|\bar{x}}=\delta_{x}$. In this case, *A* is spectral with respect to the family of states $\eta_{1|\bar{x}}$, and the hypotheses of Lemma 1 are satisfied.

Here is another, superficially quite different, way of arriving at spectrality. Suppose *A* has a conjugate, $\bar{A}$. Call a transformation Φ symmetric with respect to $\eta_{A}$ iff, for all x,y∈X(A),

$$\eta_{A}\left(\Phi^{*}x,\bar{y}\right)\;=\;\eta_{A}\left(x,{\bar{\Phi }}^{*}y\right).$$

Say that a state α is preparable by a filter Φ iff α=Φ(ρ), where ρ is the maximally-mixed state.

**Lemma** **3.** Let A have a conjugate, $\bar{A}$, and suppose every state of A is preparable by a symmetric filter. Then, A is spectral.

**Proof.**  Let α=Φ(ρ) where Φ is a filter on a test E∈M(A), say $\Phi \left(x\right)=t_{x}x$ for all x∈E. Then:

$$\;\alpha =\Phi \left({\hat{\eta }}^{*}\left(\bar{u}\right)\right)=\eta \left(\Phi^{*}\left(\cdot \right),\bar{u}\right)=\eta \left(\;\cdot \;,{\bar{\Phi }}^{*}\left(\bar{u}\right)\right)=\sum_{x\in E}\eta \left(\;\cdot \;,t_{x}\bar{x}\right)=\sum_{x\in E}t_{x}\frac{1}{n}\delta_{x}.$$

 ☐

Thus, the hypotheses of either Corollary 2 or Lemma 3 will supply the needed spectral assumption that makes Lemma 1 work (in fact, it is not hard to see that these hypotheses are actually equivalent, an exercise I leave for the reader).

To obtain a Jordan model, we still need homogeneity. This is obviously implied by the preparability condition in Lemma 3, provided the preparing filters Φ can be taken to be reversible whenever the state to be prepared is non-singular. On the other hand, as noted above, in the presence of spectrality, it is enough to have arbitrary reversible filters, as these allow one to prepare the spectral decompositions of arbitrary non-singular states. Thus, conditions (a) and (b) below both imply that *A* is a Jordan model. Conversely, one can show that any Jordan model satisfies both (a) and (b), closing the loop [17]:

**Theorem** **3.** *The following are equivalent:*

*(a)*  A has a conjugate, and every non-singular state can be prepared by a reversible symmetric filter;
*(b)*  A is sharp, has a conjugate, satisfies the correlation condition and has arbitrary reversible filters;
*(c)*  A is a Jordan model.

## 5. Measurement, Memory and Correlation

Of the spectrality-underwriting conditions given in Lemmas 2 and 3, the one that seems less transparent (to me, anyway) is the correlation condition, i.e., that every state arises as the marginal of a correlating bipartite state. While surely less ad hoc than spectrality, this still calls for further explanation. Suppose we hope to implement a measurement of a test E∈M(A) dynamically. This would involve bringing up an ancilla system *B* (also uniform, suppose; and which we can suppose, by suitable coarse-graining, if necessary, to have tests of the same cardinality as *A*’s) in some “ready” state $\beta_{o}$. We would then subject the combined system AB to some physical process, at the end of which, AB is in some final joint state ω, and *B* is (somehow!) in one of a set of record states, $\beta_{x}$, each corresponding to an outcome x∈X(A). (This way of putting things takes us close to the usual formulation of the quantum-mechanical “measurement problem”, which I certainly do not propose to discuss here. The point is only that, if any dynamical process, describable within the theory, can account for measurement results, it should be consistent with this description.)

We would like to insist that:

(a) The states $\beta_{x}$ are distinguishable, or readable, by some test F∈M(B). This means that for each x∈E, there is a unique y∈F such that $\beta_{x}\left(y\right)=1$. Note that this sets up an injection f:E→F.
(b) The record states must be accurate, in the sense that if we were to measure *E* on *A*, and secure x∈E, the record state $\beta_{x}$ should coincide with the conditional state $\omega_{2|x}$ (if this is not the case, then a measurement of *A* cannot correctly calibrate the system *B* as a measuring device for *E*).

It follows from (a) and (b) that, for x∈E and y≠f(x)∈F,

$$\omega \left(x,y\right)=\omega_{1}\left(x\right)\omega_{2|x}\left(y\right)=\omega_{1}\left(x\right)\beta_{x}\left(y\right)=0.$$

In other words, ω must correlate *E* with *F*, along the bijection $f:E\to F_{o}\subseteq F$. If the measurement process leaves α undisturbed, in the sense that $\omega_{1}=\alpha$, then α dilates to a correlating state. This suggests the following *non-disturbance principle:* every state can be measured, by some test E∈M(A), without disturbance. Lemma 2 then tells us that if *A* is sharp and satisfies the non-disturbance principle, every state of *A* is spectral.

Here is a slightly different, but possibly more compelling, version of this story. Suppose we can perform a test *E* on *A* directly (setting aside, that is, any issue of whether or not this can be achieved through some dynamical process): this will result in an outcome *x* occurring. To do anything with this, we need to record its having occurred. This means we need a storage medium, *B* and a family of states $\beta_{x}$, one for each x∈E, such that if, on performing the test *E*, we obtain *x*, then *B* will be in state $\beta_{x}$. Moreover, these record states need to be readable at a later time, i.e., distinguishable by a later measurement on *B*. To arrange this, we need *A* and *B* to be in a joint state, associated with a joint probability weight ω, such that $\omega_{1}=\alpha$ (because we want to have prepared *A* in the state α) and $\beta_{x}=\omega_{2|x}$ for every x∈E. We then measure *E* on *A*; upon our obtaining outcome x∈E, *B* is in the state $\beta_{x}$. Since the ensemble of states $\beta_{x}$ is readable by some F∈M(B) with |F|≥|E|, we have correlation, and α must also be spectral.

Of course, these desiderata cannot always be satisfied. What is true, in QM, is that for every choice of state α, there will exist some test that is recordable in that state, in the foregoing sense. If we promote this to the general principle, we again see that every state is the marginal of a correlating state, and hence spectral, if *A* is sharp.

## 6. Composites and Categories

Thus far, we have been referring to the correlator $\eta_{A}$ as a joint state, but dodging the question: *state of what?* Mathematically, nothing much hangs on this question: it is sufficient to regard $\eta_{A}$ as a bipartite probability assignment on *A* and $\bar{A}$. However, it would surely be more satisfactory to be able to treat it as an actual physical state of some composite system $A\bar{A}$. How should this be chosen? As mentioned above, one possibility is to take $A\bar{A}$ to be the maximal tensor product of the models *A* and $\bar{A}$ [24]. By definition, this has for its states all non-signaling probability assignments with conditional states belonging to *A* and $\bar{A}$. However, we might want composite systems, in particular $A\bar{A}$, to satisfy the same conditions we are imposing on *A* and $\bar{A}$, i.e., to be a Jordan model. If so, we need to work somewhat harder: the maximal tensor product will be self-dual only if *A* is classical.

In order to be more precise about all this, the first step is to decide what ought to count as a composite of two probabilistic models. If we mean to capture the idea of two physical systems that can be acted upon separately, but which cannot influence one another in any observable way (e.g., two spacelike-separated systems), the following seems to capture the minimal requirements:

**Definition** **13.** A non-signaling composite of models A and B is a model AB, together with a mapping $\pi :X\left(A\right)\times X\left(B\right)\to V^{*}{\left(AB\right)}_{+}$ such that:

$$\sum_{x\in E,y\in F}\pi \left(x,y\right)=u_{AB}$$

*and, for ω∈Ω(AB), ω∘π is a joint state on A and B, as defined in Section 2.*

The idea here, expressed in Alice-and-Bob language (Alice controlling system *A*, Bob controlling system *B*), is that π(x,y) is an effect of the composite system AB, corresponding to *x* being observed by Alice and *y*, by Bob. In many cases, π(x,y) will actually be an outcome in X(AB). Indeed, we usually have π:X(A)×X(B)→X(AB) injective, and for E∈M(A),F∈M(B), π(E×F)={π(x,y)|x∈E,y∈F} a test in M(AB). The rank of AB will then be the product of the ranks of *A* and *B*. Accordingly, let us call a non-signaling composite with these these properties *multiplicative*. Composites in real and complex quantum mechanics are multiplicative; in quaternionic quantum mechanics, with the most plausible definition of tensor product, they are not [28].

Therefore, the question becomes: can one construct, for Jordan models *A* and *B*, a non-signaling composite AB that is also a Jordan model? At present, and in this generality, this question seems to be open, but some progress is made in [28]: if neither *A*, nor *B* contain the exceptional Jordan algebra as a summand, such a composite can indeed be constructed, and in multiple ways. Moreover, under a considerably more restrictive definition of “Jordan composite”, no Jordan composite AB can exist if either factor has an exceptional summand.

**Categories of Self-Dual Probabilistic Models.** It is natural to interpret a physical theory as a category, in which objects represent physical systems and morphisms represent physical processes having these systems (or their states) as inputs and outputs. In order to discuss composite systems, this should be a symmetric monoidal category. That is, for every pair of objects A,B, there should be an object A⊗B, and for every pair of morphisms $f:A\to A^{\prime }$ and $g:B\to B^{\prime }$, there should be a morphism $f⊗g:A⊗B\to A^{\prime }⊗B^{\prime }$, representing the two processes *f* and *g* occurring “in parallel”. One requires that ⊗ be associative and commutative, and have a unit object *I*, in the sense that there exist canonical isomorphisms $\alpha_{A,B;C}:A⊗\left(B⊗C\right)≃\left(A⊗B\right)⊗C$, $\sigma_{A,B}:A⊗B≃B⊗A$, $\lambda_{A}:I⊗A≃A$ and $\rho_{A}:A⊗I\to A$/ These must satisfy various “naturality conditions”, guaranteeing that they interact correctly; see [29] for details. One also requires that ⊗ be bifunctorial, meaning that ${id}_{A}⊗{id}_{B}={id}_{A⊗B}$, and if $f:A\to A^{\prime }$, $f^{\prime }:A^{\prime }\to A^{\prime \prime }$, $g:B\to B^{\prime }$ and $g^{\prime }:B^{\prime }\to B^{\prime \prime }$, then:

$$\left(f^{\prime }⊗g^{\prime }\right)\circ \left(f⊗g\right)=\left(f^{\prime }\circ f\right)⊗\left(g^{\prime }\circ g\right).$$

By a *probabilistic theory*, I mean a category of probabilistic models and processes; that is, objects of C are models, and a morphism A→B, where A,B∈C, is a process V(A)→V(B). A *monoidal probabilistic theory* is such a category, C, carrying a symmetric monoidal structure A,B↦AB, where AB is a non-signaling composite in the sense of the definition above. I also assume that the monoidal unit, *I*, is the trivial Model 1 with V(1)=R, and that, for all A∈C,

(a) α∈Ω(A) iff the mapping α:R→V(A) given by α(1)=α belongs to C(I,A);
(b) The evaluation functional $\hat{x}$ belongs to C(A,I) for all outcomes x∈X(A).

Call C *locally tomographic* iff AB is a locally tomographic composite for all A,B∈C. Much of the qualitative content of (finite-dimensional) quantum information theory can be formulated in purely categorical terms [11,18,30]. In particular, in the work of Abramsky and Coecke [18], it is shown that a range of quantum phenomena, notably gate teleportation, is available in any *dagger-compact* category. For a review of this notion, as well as a proof of the following result, see Appendix D:

**Theorem** **4.** *Let C be a locally-tomographic monoidal probabilistic theory, in which every object A∈C is sharp, spectral and has a conjugate $\bar{A}\in C$, with $\eta_{A}\in \Omega \left(A\bar{A}\right)$. Assume also that, for all A,B∈C,*

*(i)*  $\bar{\bar{A}}=A$, with $\eta_{\bar{A}}\left(\bar{a},b\right)=\eta_{A}\left(a,\bar{b}\right)$;
*(ii)*  If ϕ∈C(A,B), then $\bar{\phi }\in C\left(\bar{A},\bar{B}\right)$.

*Then, C has a canonical dagger-compact structure, in which $\bar{A}$ is the dual of A with $\eta_{A}:R\to V\left(A\bar{A}\right)$ as the co-unit.*

**Jordan composites.** The local tomography assumption in Theorem 4 is a strong constraint. As is well known, the standard composite of two real quantum systems is not locally tomographic, yet the category of finite-dimensional real mixed-state quantum systems is certainly dagger-compact and satisfies the other assumptions of Theorem 4, so local tomography is definitely not a necessary condition for dagger-compactness.

This raises some questions. One is whether local tomography can simply be dropped in the statement of Theorem 4. At any rate, at present, I do not know of any non-dagger-compact monoidal probabilistic theory satisfying the other assumptions.

Another question is whether there exist examples other than real QM of non-locally-tomographic, but still dagger-compact, monoidal probabilistic theories satisfying the assumptions of Theorem 2. The answer to this is yes. Without going into detail, the main result of [28] is that one can construct a dagger-compact category in which the objects are Hermitian parts of finite-dimensional real, complex and quaternionic matrix algebras, that is the Euclidean Jordan algebras corresponding to finite-dimensional real, complex or quaternionic quantum-mechanical systems, and morphisms are certain completely positive mappings between enveloping complex *-algebras for these Jordan algebras. The monoidal structure gives *almost* the expected results: the composite of two real quantum systems is the real system corresponding to the usual (real) quantum-mechanical composite of the two components (and, in particular, is not locally tomographic). The composite of two quaternionic systems is a real system (see [11] for an account of why this is just what one wants). The composite of a real and a complex, or a quaternionic and a complex, system is again complex. The one surprise is that the composite of two standard complex quantum systems, in this category, is not the usual thing, but rather, comes with an extra superselection rule. This functions to make time-reversal a legitimate physical operation on complex systems, as it is for real and quaternionic systems. This is part of the price one pays for the dagger-compactness of this category.

## 7. Conclusions

As promised, we have here an easy derivation of something close to orthodox, finite-dimensional QM, from operationally or probabilistically transparent assumptions. As discussed earlier, this approach offers, in addition to its relative simplicity, greater latitude than the locally-tomographic axiomatic reconstructions of [7,8,9,10], putting us in the slightly less constrained realm of formally real Jordan algebras. This allows for real and quaternionic quantum systems, superselection rules and even theories, such as the ones discussed in Section 6, in which real, complex and quaternionic quantum systems coexist and interact.

There remains some mystery as to the proper interpretation of the conjugate system $\bar{A}$. Operationally, the situation is clear enough: if we understand *A* as controlled by Alice and $\bar{A}$, by Bob, then if Alice and Bob share the state $\eta_{A}$, then they will always obtain the same result, as long as they perform the same test. However, what does it mean physically that this should be possible (in a situation in which Alice and Bob are still able to choose their tests independently)? In fact, there is little consensus (that I can find, anyway) among physicists as to the proper interpretation of the conjugate of the Hilbert space representing a given quantum-mechanical system. One popular idea is that the conjugate is a time-reversed version of the given system; but why, then, should we expect to find a state that perfectly correlates the two? At any rate, finding a clear physical interpretation of conjugate systems, even (or especially!) in orthodox quantum mechanics, seems to me an urgently important problem.

I would like to close with another problem, this one of mainly mathematical interest. The hypotheses of Theorem 2 yield a good deal more structure than just a homogeneous, self-dual cone. In particular, we have a distinguished set M(A) of orthonormal observables in $V^{*}\left(A\right)$, with respect to which every effect has a spectral decomposition. Moreover, with a bit of work, one can show that this decomposition is essentially unique. More exactly, if $a=\sum_{i}t_{i}p_{i}$ where the coefficients $t_{i}$ are all distinct and the effects $p_{1},\ldots ,p_{k}$ are associated with a coarse-graining of a test E∈M(A), then both the coefficients and the effects are uniquely determined. The details are in Appendix B. Using this, we have a functional calculus on $V^{*}\left(A\right)$, i.e., for any real-valued function *f* of a real variable and any effect *a* with spectral decomposition $\sum_{i}t_{i}p_{i}$ as above, we can define $f\left(a\right)=\sum_{i}f\left(t_{i}\right)p_{i}$. This gives us a unique candidate for the Jordan product of effects *a* and *b*, namely,

$$a\cdot b=\frac{1}{2}\left({\left(a+b\right)}^{2}-a^{2}-b^{2}\right)).$$

We know from Theorem 2 (and thus, ultimately, from the KV theorem) that this is bilinear. The challenge is to show this without appealing to the KV theorem (the fact that the state spaces of “bits” are always balls, as shown in Appendix C, is perhaps relevant here).

## Figures and Tables

**Table 1:** A joint state for two square bits.

	x	x’	y	y’
x	1/2	0	1/2	0
x’	0	1/2	0	1/2
y	0	1/2	1/2	0
y’	1/2	0	0	1/2

## Appendix A. Models with Symmetry

Recall that a probabilistic model *A* is sharp iff, for every measurement outcome x∈X(A), there exists a unique state $\delta_{x}\in \Omega \left(A\right)$ with $\delta_{x}\left(x\right)=1$. While this is clearly a very strong condition, it is not an unreasonable one. In fact, given the test space M(A), we can often choose the state space Ω(A) in such a way as to guarantee that *A* is sharp. In particular, this is the case when M(A) enjoys enough symmetry.

**Definition** **A1.** Let G be a group. A G-test space is a test space (X,M) where X is a G-space, that is, where X comes equipped with a preferred G-action G×X→X, (g,x)↦gx, such that gE∈M for all E∈M. A G-model is a probabilistic model A such that (i) M(A) is a G-test space and (ii) Ω(A) is invariant under the action of G on probability weights given by $\alpha \mapsto g\alpha :=\alpha \circ g^{-1}$ for g∈G.

**Lemma** **A1.** Let A be a finite-dimensional G-model, and suppose G acts transitively on the outcome space X(A). Suppose also that A is unital, i.e., for every x∈X(A), there exists at least one state α with α(x)=1. Then, there exists a G-invariant convex subset Δ⊆Ω(A) such that $A^{\prime }=\left(M\left(A\right),\Delta \right)$ is a sharp G-model.

**Proof.** For each x∈X(A), let $F_{x}$ denote the face of Ω(A) consisting of states α with α(x)=1. Let $\beta_{x}$ be the barycenter of $F_{x}$. It is easy to check that $F_{gx}=gF_{x}$ for every g∈G. Thus, $g\beta_{x}=\beta_{gx}$, i.e., the set of barycenters $\beta_{x}$ is an orbit. Let Δ be the convex hull of these barycenters. Then, Δ is invariant under *G*. If α∈Δ with α(x)=1, then $\alpha \in F_{x}\cap \Delta =\left\{\beta_{x}\right\}$, so (M(A),Δ) is sharp. ☐

## Appendix B. Uniqueness of Spectral Decompositions

Let *A* be a model satisfying the conditions of Lemma 1. In particular, every $a\in E\left(A\right)=V^{*}\left(A\right)$ has a spectral representation $a=\sum_{x\in E}t_{x}\hat{x}$ for some test E∈M(A). In general, this expansion is highly non-unique. For instance, the unit $u_{A}$ can be expanded as $\sum_{x\in E}\hat{x}$ for any test E∈M(A). The aim in this Appendix is to obtain a form of spectral expansion for effects that is unique.

Call a subset of a test an *event*. That is, D⊆X(A) is an event iff there exists a test E∈M(A) with D⊆E. The probability of an effect *D* in a state α is $\alpha \left(D\right)=\sum_{x\in E}\alpha \left(x\right)$. Thus, any event gives rise to an effect, $\hat{D}$, given by $\hat{D}\left(\alpha \right)=\alpha \left(D\right)$. Evidently,

$$\hat{D}\;:=\;\sum_{x\in E}\hat{x}.$$

A test is a maximal event, and for any test E∈M(A), $\hat{D}=u$.

**Definition** **A2.** An effect $p\in V^{*}\left(A\right)$ is sharp iff it has the form $p=\hat{D}$ for some event D. A set of sharp effects $p_{1},\ldots ,p_{n}\in V^{*}\left(A\right)$ is jointly orthogonal with respect to M(A) iff there exists a test E∈M(A) and pairwise disjoint events $D_{1},\ldots ,D_{n}\subseteq E$ with $p_{i}={\hat{D}}_{i}$ for i=1,…,n.

Given an arbitrary element $a\in V^{*}\left(A\right)$ with spectral decomposition $a=\sum_{x\in E}t_{x}\hat{x}$, we can isolate distinct values $t_{o}>t_{1}>\ldots >t_{k}$ of the coefficients $t_{x}$. Letting $E_{i}=\left\{x\in E|t_{x}=t_{i}\right\}$ and setting $p_{i}=p\left(E_{i}\right)=\sum_{x\in E_{i}}\hat{x}$, we have $a=\sum_{i}t_{i}p_{i}$, with $p_{1},\ldots ,p_{n}$ jointly orthogonal. Suppose there is another such decomposition, say $a=\sum_{j}s_{j}q_{j}$, with $q_{j}={\hat{F}}_{j}=\sum_{y\in F_{j}}\hat{y}$, where $F_{1},\ldots ,F_{l}\subseteq F\in M\left(A\right)$ are pairwise disjoint, and again, with the coefficients in descending order, say $s_{0}>s_{1}>\cdots >s_{l}$.

**Lemma** **A2.** In the situation described above, $t_{0}=s_{0}$ and $p_{0}=q_{0}$.

**Proof.** Normalize the inner product on E(A) so that ∥x∥=1 for all outcomes *x*. Then, for any sharp effect $p=\hat{D}$, *D* an event, we have ${∥D∥}^{2}=\left|D\right|$, the cardinality of *D*. Choosing any outcome $x_{0}\in E_{0}$, set $\alpha =|x_{0}\rangle$, i.e., $\alpha \left(\hat{x}\right)=\langle \hat{x},{\hat{x}}_{0}\rangle$ for all x∈X(A). Then, α∈Ω(A), $\alpha (p_{0})=1$ and $\alpha (p_{i})=0$ for i>0. Thus,

$$t_{0}=\alpha \left(a\right)=\sum_{j}s_{j}\alpha \left(q_{j}\right).$$

Since the coefficients $\alpha (q_{j})$ are sub-convex, the right-hand side is no larger than the largest of the values $s_{j}$, namely, $s_{o}$. Thus, $t_{0}\leq s_{0}$. The same argument, with the roles of the two decompositions reversed, shows that $s_{0}\leq t_{0}$. Thus, $s_{0}=t_{0}$. Now again, let $x\in E_{0}$: then,

$$\langle \hat{x},p_{0}\rangle =\sum_{y\in E_{0}}\langle \hat{x},\hat{y}\rangle =\langle x,x\rangle =1,$$

whence, $\langle \hat{x},a\rangle =t_{o}$. However, we then have (using the fact that $s_{0}=t_{0}$):

$$t_{0}=\langle \hat{x},a\rangle =\langle \hat{x}\;,\;t_{0}q_{0}+\sum_{j=1}^{l}s_{j}q_{j}\rangle =t_{o}\langle \hat{x},q_{0}\rangle +\sum_{j=1}^{l}s_{j}\langle \hat{x},q_{j}\rangle .$$

Since $\sum_{j=0}^{l}\langle \hat{x},q_{j}\rangle \leq \langle \hat{x},u\rangle =\leq 1$, the sum in the last expression above is a sub-convex combination of the distinct values $s_{o}>\cdots >s_{l}$. This can equal $t_{0}=s_{0}$, the maximum of these values, only if $\langle \hat{x},q_{0}\rangle =1$ and $\langle \hat{x},q_{j}\rangle =0$ for the remaining $q_{j}$. It follows that $\langle p_{0},q_{0}\rangle =\sum_{x\in E_{0}}\langle \hat{x},q_{0}\rangle =|E_{0}|=∥p_{0}{∥}^{2}$. The same argument, with *p*’s and *q*’s interchanged, shows that $\langle p_{0},q_{0}\rangle ={∥q_{0}∥}^{2}$. Hence, $∥p_{0}∥=∥q_{0}∥$, and $\langle p_{0},q_{0}\rangle =∥p_{0}{∥}^{2}=∥p_{0}∥∥q_{0}∥$, whence, $p_{0}=q_{0}$. ☐

**Proposition** **A1.** Every $a\in V^{*}\left(A\right)$ has a unique expansion of the form $a=\sum_{i=0}^{k}t_{i}p_{i}$ where $t_{0}>t_{1}>\ldots >t_{k}$ are non-zero coefficients and $p_{1},\ldots ,p_{n}$ are jointly orthogonal sharp effects.

**Proof.** Suppose $a=\sum_{i=1}^{k}t_{i}p_{i}$, as above, and also $a=\sum_{j=1}^{l}s_{j}q_{j}$, with $s_{0}>\cdots >s_{l}>0$ and $q_{j}$ pairwise orthogonal sharp effects. We shall show that k=l, and that $t_{i}=s_{i}$ and $p_{i}=q_{i}$ for each i=1,…,k. Lemma A2 tells us that $t_{0}=s_{0}$ and $p_{0}=s_{0}$. Hence,

$$\sum_{i=1}^{k}t_{i}p_{i}=a-t_{o}p_{o}=a-s_{0}q_{0}=\sum_{j=1}^{l}s_{j}q_{j}.$$

Applying Lemma A2 recursively, we find that $t_{i}=s_{i}$ and $p_{i}=q_{i}$ for i=1,…,min(k,l). If k≠l, say k<l; we then have:

$$t_{k}p_{k}=s_{k}q_{k}+\sum_{j=k+1}^{l}s_{j}q_{j}=t_{k}p_{k}+\sum_{j=k+1}^{l}s_{j}q_{j}$$

whence, $\sum_{j=k+1}^{l}s_{j}q_{j}=0$, which is impossible since all $q_{j}$ are sharp and the coefficients $s_{j}$ are strictly positive. Hence, l=k, and the proof is complete. ☐

## Appendix C. Bits Are Balls

In most other reconstructions of QM [8,9,10], the first step is to show that the state space of a bit, that is, a system in which every state is the mixture of two sharply-distinguishable pure states, is a ball. In our approach, this fact is an easy consequence of Lemma 1. In our framework, we will define a *bit* to be a sharp, uniform model *A* with rank two, in which every state has the form $t\delta_{x}+\left(1-t\right)\delta_{x^{\prime }}$, where $\left\{x,x^{\prime }\right\}\in M\left(A\right)$. Note that this implies that *A* is spectral.

**Lemma** **A3.** Let A be a bit with conjugate $\bar{A}$. Then, Ω(A) is a Euclidean ball, the extreme points of which are the states $\delta_{x}$, x∈X(A).

**Proof.** By Lemma 1, E(A) carries a self-dualizing inner product such that $\langle \hat{x},\hat{y}\rangle =0$ for {x,y}∈M(A), and which we can normalize so that $∥\hat{x}∥=1$ for each outcome x∈X(A), so that $\langle u,\hat{x}\rangle =\langle \hat{x},\hat{x}\rangle =1$ and ${∥u∥}^{2}=2$. Every state α∈Ω(A) corresponds to a unique vector $a\in E{\left(A\right)}_{+}$ with 〈a,u〉=1, where $\alpha \left(x\right)=\langle a,\hat{x}\rangle$ for all x∈X(A); conversely, every vector $a\in E{\left(A\right)}_{+}$ with 〈a,u〉=1 corresponds in this way to a state. In particular, the state $\delta_{x}$ corresponds to the unit vectors $\hat{x}$, and the maximally-mixed state corresponds to the vector $\frac{1}{n}u$. To simplify the notation, let us agree for the moment to write ρ for this vector. Thus, $\langle \rho ,\hat{x}\rangle =\frac{1}{2}$, ${∥\rho ∥}^{2}=\frac{1}{4}\langle u,u\rangle =\frac{1}{2}$, and hence,

$$∥\rho -\hat{x}{∥}^{2}={∥\rho ∥}^{2}-2\langle \rho ,\hat{x}\rangle +{∥\hat{x}∥}^{2}=\frac{1}{2}.$$

Thus, $\hat{X}\left(A\right):=\left\{\;\hat{x}\;|\;x\in X\left(A\right)\;\right\}$ lies on the sphere of radius $1/\sqrt{2}$ about the state ρ. I now claim that any a∈E(A) with 〈a,u〉=1 (in effect, any state) such that $∥\rho -a∥\leq 1/\sqrt{2}$ belongs to the positive cone $E{\left(A\right)}_{+}$. To see this, use spectrality to decompose *a* as $s\hat{x}+t\hat{y}$ where {x,y}∈M(A) and s,t∈R. Consider now the two-dimensional subspace $E_{x,y}$ spanned by $\hat{x}$ and $\hat{y}$. With respect to the inner product inherited from E, we can regard this as a two-dimensional Euclidean space, in which *a* is represented by the Cartesian coordinate pair (s,t). Expanding ρ as $\rho =\frac{1}{2}\left(\hat{x}+\hat{y}\right)$, we see that $\rho \in E_{x,y}$ with coordinates (1/2,1/2). The point (t,s) lies, therefore, in the disk of radius $1/\sqrt{2}$ centered at (1/2,1/2) in $E_{x,y}$. Moreover, as 〈a,u〉=1, we see that s+t=1, i.e., (s,t) lies on the line of slope −1 through (1/2,1/2). This puts (s,t) in the positive quadrant of this plane, i.e., s≥0 and t≥0. However, then $a\in E{\left(A\right)}_{+}$, as claimed. ☐

It follows that, for rank-two models, we do not even need to invoke homogeneity: they all correspond to spin factors. Letting *d* denote the dimension of the state space (that is, d=dim(E)−1), we see that if d=1, we have the classical bit; d=2 gives the real quantum-mechanical bit, d=3 gives the familiar Bloch sphere, i.e., the usual qubit of complex QM; while d=5 corresponds to the quaternionic unit sphere, giving us the quaternionic bit. The generalized bits with d=4 and d≥6 are more exotic “post-quantum” possibilities.

## Appendix D. Locally-Tomographic and Dagger-Compactness

A *dagger* on a category C is a contravariant functor †:C→C that is the identity on objects and satisfies $†\circ †={id}_{C}$. That is, if $A\overset{f}{⟶}B$ is a morphism in C, then $A\overset{f^{†}}{⟵}B$, with $f^{††}=f$ and ${\left(f\circ g\right)}^{†}=g^{†}\circ f^{†}$ whenever f∘g is defined. An isomorphism f:A≃B in C is then said to be *unitary* iff $f^{†}=f^{-1}$. One says that C is *†-monoidal* iff C is equipped with a symmetric monoidal structure ⊗ such that ${\left(f⊗g\right)}^{†}=f^{†}⊗g^{†}$, and such that the canonical isomorphisms $\alpha_{A,B,C}$, $\sigma_{A,B}$, $\lambda_{A}$ and $\rho_{A}$ are all unitary.

A *dual* for an object *A* in a symmetric monoidal category C is a structure $\left(A^{\prime },\eta ,\epsilon \right)$ where $A^{\prime }\in C$ and $\eta :I\to A⊗A^{\prime }$ and $\epsilon :A^{\prime }⊗A\to I$, such that:

$$\left({id}_{A}⊗\epsilon \right)\circ \left(\eta ⊗{id}_{A}\right)={id}_{A}\;\;and\;\;\left(\epsilon ⊗{id}_{A^{\prime }}\right)\circ \left({id}_{A^{\prime }}⊗\eta \right)={id}_{A^{\prime }}$$

up to the natural associator and unit isomorphisms. If C is †-monoidal and $\epsilon =\sigma_{A,A^{\prime }}\circ \eta_{A}^{†}$, then $\left(A^{\prime },\eta ,\epsilon \right)$ is a *dagger-dual*. A category in which every object *A* has a specified dual $\left(A^{\prime },\eta_{A},\epsilon_{A}\right)$ is compact closed, and a dagger-monoidal category in which every object has a given dagger-dual is dagger-compact. See [18,30] for details.

An important example of all this is the category ${\mathrm{FdHilb}}_{R}$ of finite-dimensional real Hilbert spaces and linear mappings. If H and K are two such spaces and ϕ:H→K, let $\phi^{†}$ be the usual adjoint of ϕ with respect to the given inner products. Letting H⊗K be the usual tensor product of H and K (in particular, with 〈x⊗y,u⊗v〉=〈x,u〉〈y,v〉 for x,u∈H and y,v∈K), ${\mathrm{FdHilb}}_{R}$ is a dagger-monoidal category with R as the monoidal unit.

Since any $H\in {\mathrm{FdHilb}}_{R}$ is canonically isomorphic to its dual space, we have also a canonical isomorphism $H⊗H≃H^{*}⊗H=\left(H\right)$ and a canonical trace functional ${Tr}_{H}:H⊗H\to R$, uniquely defined by ${Tr}_{H}\left(x⊗y\right)=\langle x,y\rangle$ for all x,y∈H. Taking $H^{\prime }=H$, let $\eta_{H}\in H⊗H$ be given by $\eta_{H}=\sum_{i}x_{i}⊗x_{i}$, where the sum is taken over any orthonormal basis $\left\{x_{i}\right\}$ for H; then, for any a∈H⊗H, $\langle \eta_{A},a\rangle =Tr\left(a\right)$. It is routine to show that ${Tr}_{H}=\sigma_{H,}\circ \eta_{H}^{†}$, so that $\eta_{H}$ and ${Tr}_{H}$ make H its own dagger-dual.

In any compact closed symmetric monoidal category C, every morphism ϕ:A→B yields a dual morphism $\phi^{\prime }:B^{\prime }\to A^{\prime }$ defined by:

$$\phi^{\prime }=\left({id}_{A^{\prime }}⊗\epsilon_{B}\right)\circ \left({id}_{A^{\prime }}⊗f⊗{id}_{B^{\prime }}\right)\circ \left(\eta_{A}⊗{id}_{B^{\prime }}\right).$$

(again, suppressing associators and left and right units). For ϕ:H→K in ${\mathrm{FdHilb}}_{R}$, one has, for any v∈A,

$$\phi^{\prime }\left(v\right)=\sum_{x\in M}\langle v,f\left(x\right)\rangle x=\sum_{x\in M}\langle f^{†}\left(v\right),x\rangle x=f^{†}\left(v\right),$$

i.e., $\phi^{\prime }=\phi^{†}$.

Now, let C be a monoidal probabilistic theory; that is, a category of probabilistic models and processes, with a symmetric monoidal structure A,B↦AB, where AB is a (non-signaling) composite in the sense discussed in Section 6. Let C is multiplicative, so that for A,B∈C, we have $\pi_{AB}:X\left(A\right)\times X\left(B\right)\to X\left(AB\right)$. Henceforward, I will write x⊗y for π(x,y) where x∈X(A) and y∈X(B). I will further assume that C’s tensor unit is I=R, and that:

(a) Every A∈C has a conjugate, $\bar{A}\in C$, with $\bar{\bar{A}}=A$;
(b) For all A,B∈C and ϕ∈C(A,B), $\bar{\phi }\in C\left(\bar{A},\bar{B}\right)$;
(c) $\bar{\bar{A}}=A$, with $\eta_{\bar{A}}\left(\bar{a},b\right):=\eta_{A}\left(a,\bar{b}\right)$.

**Remark** **A1.** (1) The chosen conjugate $\bar{A}$ for A∈C required by Condition (a) is equipped with a canonical isomorphism $\gamma_{A}:A≃\bar{A}$, with $\bar{x}=\gamma \left(x\right)$ for every x∈X(A). As discussed in Section 4, this extends to an order-isomorphism $E\left(A\right)≃E\left(\bar{A}\right)$, which we again write as $\gamma_{A}\left(a\right)=\bar{a}$ for a∈E(A). Notice, however, that $\gamma_{A}$ is not assumed to be a morphism in C. (2) In spite of this, Condition (b) requires that $\bar{\phi }=\gamma_{B}\circ \phi \circ \gamma_{A}^{-1}$ does belong to $C(\bar{A},\bar{B})$ for ϕ∈C(A,B). Notice here that $\phi \mapsto \bar{\phi }$ is functorial. *(3) The second part of Condition (c) is redundant if every model A in C is sharp (since in this case, there is at most one correlator between $\bar{A}$ and A). Notice, too, that Condition (c) implies that:*

$$\langle \bar{x},\bar{y}\rangle =\eta_{\bar{A}}\left(\bar{x},y\right)=\eta_{A}\left(x,\bar{y}\right)=\langle \hat{x},\hat{y}\rangle$$

*for all x,y∈E(A).*

We are now ready to prove Theorem 4. We continue to assume that C is a locally-tomographic, multiplicative monoidal probabilistic theory, satisfying Conditions (a), (b) and (c) above. We wish to show that if every A∈C is sharp and spectral, then C has a canonical dagger, with respect to which it is dagger-compact.

Before proceeding, it will be convenient to dualize our representation of morphisms, so that ϕ∈C(A,B) means that ϕ is a positive linear mapping E(B)→E(A) (thus, our co-unit $\eta \in C(I,A⊗A^{\prime })$ becomes a positive linear mapping $\eta_{A}:E\left(A⊗A^{\prime }\right)\to R$, and similarly, a unit $\epsilon_{A}\in C\left(A^{\prime }⊗A,I\right)$ becomes a positive linear mapping $R\to E(A^{\prime }⊗A)$, i.e, an element of $E(A⊗A^{\prime })$). By Lemma 1, for every A∈C, the space E(A) carries a canonical self-dualizing inner product ${\langle \;,\;\rangle }_{A}$, with respect to which E(A)≃V(A).

**Lemma** **A4.** For all models A,B∈C, the inner product on E(AB) factors, in the sense that if u,x∈E(A) and v,y∈E(B), then 〈u⊗v,x⊗y〉=〈u,x〉〈v,y〉.

**Proof.** This follows from the sharpness of A,B and AB. For u∈X(A), v∈X(B), let $\delta_{u},\delta_{v}$ and $\delta_{u⊗v}$ denote the unique states of *A*, *B* and AB such that $\delta_{u}\left(u\right)=\delta_{v}\left(v\right)=\delta_{u⊗v}\left(u⊗v\right)=1$. Since $\left(\delta_{u}⊗\delta_{v}\right)\left(u⊗v\right)$ is also one, we conclude that $\delta_{u⊗v}=\delta_{u}⊗\delta_{v}$. However, we also have $\delta_{u}\left(x\right)=n\langle \hat{u},\hat{x}\rangle$, $\delta_{v}\left(y\right)=m\langle \hat{v},\hat{y}\rangle$ and $\delta_{u⊗v}\left(x⊗y\right)=nm\langle \hat{u}⊗\hat{v},\hat{x}⊗\hat{y}\rangle$, where n,m and nm are the ranks, respectively, of *A*, *B* and A⊗B. This establishes the claim. ☐

It follows that C is a monoidal subcategory of ${\mathrm{FdHilb}}_{R}$. In effect, we are going to show that C inherits a dagger-compact structure from ${\mathrm{FdHilb}}_{R}$, with the minor twist that we will take $\bar{A}$, rather than *A*, as the dual for A∈C. We define the dagger of ϕ∈C(A,B) to be the Hermitian adjoint of ϕ:E(A)→E(B) with respect to the canonical inner products on E(A) and E(B). At this point, it is not obvious that $\phi^{†}$ belongs to C. In order to show that it does, we first need to show that C is compact closed. To define the unit, let $e_{A}\in E\left(\bar{A}\right)⊗E\left(\bar{A}\right)=E\left(\bar{A}A\right)$ (note the use of local tomography here) to be the vector with $\langle e_{A},\;\cdot \;\rangle =\eta_{A}$, i.e., for all a,b∈E(A),

$$\langle e_{A},\bar{a}⊗b)=\eta_{A}\left(a⊗\bar{b}\right)=\langle a,b\rangle .$$

Since $E(A\bar{A})$ is self-dual, $e_{A}\in E{\left(A\bar{A}\right)}_{+}$.

**Lemma** **A5.** With $\eta_{A}$ and $e_{A}$ defined as above, $\bar{A}$ is a dual for A for every A∈C. In particular, C is compact closed.

**Proof.** Choose an orthonormal basis M⊆E(A). Local tomography and Lemma A4 tell us that $\bar{M}⊗M=\left\{\bar{a}⊗a|a\in M\right\}$ is then an orthonormal basis for $E(\bar{A}A)$ (note here that a,b∈M are not necessarily even positive, let alone in X(A)). If we expand $e_{A}$ with respect to this basis, we have:

$$e_{A}=\sum_{a,b\in M}\langle e_{A},\bar{a}⊗b\rangle \bar{a}⊗b$$

Since the basis is orthonormal, we have:

$$\langle e_{A},\bar{a}⊗a\rangle =\langle a,a\rangle ={∥a∥}^{2}=1$$

and for a≠b, both in *M*,

$$\langle e_{A},\bar{a}⊗b\rangle =\langle a,b\rangle =0$$

Hence, $e_{A}=\sum_{a\in M}\bar{a}⊗a$. Regarding $e_{A}$ as a morphism $I\to \bar{A}⊗A$, we now have, for any v∈E(A),

$$\begin{array}{ccc} \left(\eta_{A}⊗{id}_{A}\right)\circ \left({id}_{A}⊗e_{A}\right)\left(v\right) & = & \left(\eta_{A}⊗id_{A}\right)\left(\sum_{x\in M}v⊗\bar{a}⊗a\right)\\ & = & \sum_{x\in M}\eta_{A}\left(v⊗\bar{a}\right)a\\ & = & \sum_{x\in M}\langle v,a\rangle a=v. \end{array}$$

Similarly, for $\bar{v}\in \bar{A}$,

$$\begin{array}{ccc} \left({id}_{\bar{A}}⊗\eta_{\bar{A}}\right)\circ \left(e_{\bar{A}}⊗{id}_{\bar{A}}\right)\left(\bar{v}\right) & = & \left({id}_{\bar{A}}⊗\eta_{\bar{A}}\right)\left(\sum_{a\in M}\bar{a}⊗a⊗\bar{v}\right)\\ & = & \sum_{x\in M}\bar{a}\eta_{A}\left(a,\bar{v}\right)=\sum_{a\in M}\langle a,v\rangle \bar{a}\\ & = & \sum_{a\in M}\langle \bar{v},\bar{a}\rangle \bar{a}=\bar{v}. \end{array}$$

 ☐

**Lemma** **A6.** If ϕ:E(A)→E(B) belongs to C, then so does $\phi^{†}:E\left(B\right)\to E\left(A\right)$.

**Proof.** Using the compact structure on C defined above, if ϕ:A→B, we construct the dual of $\bar{\phi }$,

$${\bar{\phi }}^{\prime }:=\left(\eta_{B}⊗{id}_{A}\right)\circ \left({id}_{B}⊗\bar{\phi }⊗{id}_{A}\right)\circ \left({id}_{B}⊗e_{A}\right):E\left(B\right)\to E\left(A\right).$$

Applying this mapping to b∈E(B), we have:

$$\begin{array}{ccc} b\mapsto \left(\eta_{B}⊗{id}_{A}\right)\left(\sum_{a\in M}b⊗\bar{\phi }\left(\bar{a}\right)⊗a\right) & = & \sum_{a\in M}\eta_{B}\left(b,\bar{\phi }\left(\bar{a}\right)\right)a.\\ & = & \sum_{a\in M}\langle b,\phi \left(a\right)\rangle a\\ & = & \sum_{a\in M}\langle \phi^{†}\left(b\right),a\rangle a=\phi^{†}\left(b\right). \end{array}$$

Thus, $\phi^{†}={\bar{\phi }}^{\prime }$, which is evidently a morphism in C. Thus, C is a dagger-, as well as a monoidal, sub-category of ${\mathrm{FdHilb}}_{R}$. Hence, the associator, swap and left- and right-unit morphisms associated with an object A∈C are all unitary (since they are unitary in ${\mathrm{FdHilb}}_{R}$), whence C is dagger-monoidal. To complete the proof of Theorem 4, we need to check that $\eta_{A}=e_{A}^{†}\circ \sigma_{A,\bar{A}}:E\left(A\bar{A}\right)\to R$. In view of our local tomography assumption, it is enough to check this on pure tensors, where a routine computation gives us $e_{A}^{†}\left(\sigma_{A,\bar{A}}\left(a⊗\bar{b}\right)\right)$ = ${\langle e_{A}^{†}\left(\bar{b}⊗a\right),1\rangle }_{1}$ = ${\langle \bar{b}⊗a,e_{A}\rangle }_{\bar{A}A}$ = 〈a,b〉 = $\eta_{A}\left(a⊗\bar{b}\right)$. ☐

**Remark** **A2.** Given that C is compact closed, with $\bar{A}$ the dual of A, the functoriality of $\phi \mapsto \bar{\phi }$ makes C strongly compact closed, in the sense of [18]. This is equivalent to dagger-compactness.

## References

[1] Von Neumann J. (1955). Mathematical Foundations of Quantum Mechanics.

[2] Schwinger J. (1959). The algebra of microscopic measurement. Proc. Natl. Acad. Sci. USA.

[3] Mackey G.W. (2004). Mathematical Foundations of Quantum Mechanics.

[4] Ludwig G. (1983). Foundations of Quantum Mechanics I.

[5] Piron C. (1978). Mathematical Foundations of Quantum Mechanics.

[6] Barnum H., Müller M., Ududec C. (2014). Higher-order interference and single-system postulates characterizing quantum theory. New J. Phys..

[7] Hardy L. (2001). Quantm theory from five reasonable axioms. arXiv.

[8] Chiribella G., D’Ariano M., Perinotti P. (2011). Informational derivation of quantum theory. Phys. Rev. A.

[9] Dakic B., Brukner C. (2009). Quantum theory and beyond: Is entanglement special?. arXiv.

[10] Masanes L., Müller M. (2011). A derivation of quantum theory from physical requirements. New J. Phys..

[11] Baez J. (2012). Division algebras and quantum theory. Found. Phys..

[12] Janotta P., Lal R. (2013). Generalized probabilistic theories without the no-restriction hypothesis. Phys. Rev. A.

[13] Faraut J., Koranyi A. (1994). Analysis on Symmetric Cones.

[14] Wilce A., Ben-Menahem Y., Hemmo M. (2012). 4.5 axioms for finite-dimensional quantum probability. Probability in Physics.

[15] Wilce A. (2011). Symmetry and composition in probabilistic theories. Electron. Notes Theor. Comput. Sci..

[16] Wilce A. (2011). Symmetry, self-duality and the Jordan structure of finite-dimensional quantum mechanics. arXiv.

[17] Wilce A. (2012). Conjugates, Filters and Quantum Mechanics. arXiv.

[18] Abramsky S., Coecke B. (2005). Abstract Physical Traces. Theor. Appl. Categories.

[19] Barnum H., Graydon M.A., Wilce A. (2015). Some nearly quantum theories. arXiv.

[20] Jordan P. (1933). Über ein Klasse nichtassoziativer hypercomplexe algebren. Nachr. Akad. Wiss. Göttingen Math. Phys. Kl. I..

[21] Von Neumann J. (1936). On an algebraic generalization of the quantum mechanical formalism (Part I). Ann. Math..

[22] Aliprantis C.D., Toukey R. (2007). Cones and Duality.

[23] Foulis D.J., Randall C.H., Neumann H. (1981). Empirical logic and tensor products. Interpretations and Foundations of Quantum Theory.

[24] Barnum H., Wilce A., Chiribella G., Spekkens R. (2016). Post-classical probability theory. Quantum Theory: Informational Foundations and Foils.

[25] Popescu S., Rohrlich D. (1994). Nonlocality as an axiom. Found. Phys..

[26] Gunson J. (1967). On the algebraic structure of quantum mechanics. Commun. Math. Phys..

[27] Chribella G., Scandolo C.M. (2015). Operational axioms for state diagonalization. arXiv.

[28] Barnum H., Graydon M., Wilce A. (2016). Composites and categories of Euclidean Jordan algebras. arXiv.

[29] Mac Lane S. (1978). Categories for the Working Mathematician.

[30] Selinger P. (2007). Dagger compact closed categories and completely positive maps. Electron. Notes Theor. Comput. Sci..

